# Sugar accumulation enhancement in sorghum stem is associated with reduced reproductive sink strength and increased phloem unloading activity

**DOI:** 10.3389/fpls.2023.1233813

**Published:** 2023-09-11

**Authors:** Xueyi Xue, Gabriel Beuchat, Jiang Wang, Ya-Chi Yu, Stephen Moose, Jin Chen, Li-Qing Chen

**Affiliations:** ^1^ Department of Energy (DOE) Center for Advanced Bioenergy and Bioproducts Innovation, University of Illinois at Urbana-Champaign, Urbana, IL, United States; ^2^ Institute for Sustainability, Energy, and Environment, University of Illinois at Urbana-Champaign, Urbana, IL, United States; ^3^ Department of Plant Biology, University of Illinois at Urbana-Champaign, Urbana, IL, United States; ^4^ Department of Crop Sciences, University of Illinois at Urbana-Champaign, Urbana, IL, United States; ^5^ Institute for Biomedical Informatics, University of Kentucky, Lexington, KY, United States

**Keywords:** sorghum, sugar accumulation, SWEETs, *Dry* locus, *bHLH093*, sink strength, phloem unloading, sugar transporter

## Abstract

Sweet sorghum has emerged as a promising source of bioenergy mainly due to its high biomass and high soluble sugar yield in stems. Studies have shown that loss-of-function *Dry* locus alleles have been selected during sweet sorghum domestication, and decapitation can further boost sugar accumulation in sweet sorghum, indicating that the potential for improving sugar yields is yet to be fully realized. To maximize sugar accumulation, it is essential to gain a better understanding of the mechanism underlying the massive accumulation of soluble sugars in sweet sorghum stems in addition to the *Dry* locus. We performed a transcriptomic analysis upon decapitation of near-isogenic lines for mutant (*d*, juicy stems, and green leaf midrib) and functional (*D*, dry stems and white leaf midrib) alleles at the *Dry* locus. Our analysis revealed that decapitation suppressed photosynthesis in leaves, but accelerated starch metabolic processes in stems. *SbbHLH093* negatively correlates with sugar levels supported by genotypes (*DD* vs. *dd*), treatments (control vs. decapitation), and developmental stages post anthesis (3d vs.10d). *D* locus gene *SbNAC074A* and other programmed cell death-related genes were downregulated by decapitation, while sugar transporter-encoding gene *SbSWEET1A* was induced. Both *SbSWEET1A* and *Invertase 5* were detected in phloem companion cells by RNA *in situ* assay. Loss of the *SbbHLH093* homolog*, AtbHLH093*, in *Arabidopsis* led to a sugar accumulation increase. This study provides new insights into sugar accumulation enhancement in bioenergy crops, which can be potentially achieved by reducing reproductive sink strength and enhancing phloem unloading.

## Introduction

Sugars produced by plants are a vital source of human nutrition and industrial biofuel production. Sugar content is tightly associated with the quality and agronomic value of sugar-rich fruits and sugar-storing crops. Maximizing the soluble sugar yield in sugar-storing crops, such as sugarcane, sugar beet, and sweet sorghum has been a subject of great interest in biofuel and sugar production. Roots of sugar beet and stems of sugarcane and sweet sorghum are the primary tissues to accumulate high levels of sugars, mainly sucrose. These tissues are sink tissues receiving photosynthetic products from source tissues through phloem transport. Phloem transport connects source-sink and sink-sink tissues, largely determining crop yield and overall biomass (Lemoine et al., 2013; Smith et al., 2018; Beuchat et al., 2020). The alteration of the source-sink relationship caused by abiotic stresses, such as drought and heat, led to serious yield loss (Fahad et al., 2017; Abdelrahman et al., 2020). The effects of sink strength manipulation have been studied in various crops, including maize, wheat, soybean, and sorghum (Patterson and Brun, 1980; Christensen et al., 1981; Heitholt and Egli, 1985; Sekhon et al., 2012; Brown and Hudson, 2017). In maize, ear removal resulted in sugar accumulation in the leaves and stems (Christensen et al., 1981; Sekhon et al., 2012). Continuous pod removal in soybean plants for 30 days led to a higher dry weight in seeds (Heitholt and Egli, 1985). Additionally, Mcalister and Krober (1958) found more nutrients in the remaining seeds and increased sugars, starch, and nitrogen in leaves and stems when a higher percentage of pods were removed from soybean plants (Mcalister and Krober, 1958), which indicates that partial removal of sink tissues benefits the remaining tissues.

Sorghum (*Sorghum bicolor* L. Moench) is a resilient C4 crop that has been domesticated for multiple uses, including food, forage, and biofuels (Vanamala et al., 2018). The adaptable nature of sorghum enables cultivation in harsh conditions, making it a vital crop in arid and semiarid regions. During sorghum breeding and domestication, sorghum has been cultivated for different uses with variable harvestable tissues such as grains and stems. Grain sorghum is grown for food, livestock feed, and liquor production since grains are rich in starch, vitamins and minerals (Hossain et al., 2022; Weiss et al., 2022). The sorghum stem has long been recognized as a source of sugar and ethanol production (Smith et al., 1987). Sweet sorghum accumulates high soluble sugar (sucrose, glucose, and fructose) in stems due to higher juice volume and higher sugar concentration (Zhang et al., 2018; Li et al., 2019). The juiciness trait is controlled in part by the *Dry* (*D*) locus (Swanson and Parker, 1931). A genome-wide association study found that the D locus is the primary QTL associated with sugar yield in sorghum, and green midribs observed from most sweet cultivars are associated with juice volume (Burks et al., 2015). Later, the corresponding gene was identified to encode a NAC transcriptional factor, SbNAC074A. Sorghum with juicy stalks and green midribs harbors a deletion or a recessive allele (*d*) encoding a loss-of-function NAC. The wild-type NAC (*D*) gene is associated with altered secondary cell wall composition and parenchyma cell death in stem pith (Casto et al., 2018; Fujimoto et al., 2018; Xia et al., 2018; Zhang et al., 2018).

To understand the variation in the spatiotemporal sugar accumulation, several transcriptomic analyses have been conducted in sorghum stem, revealing that starch turnover is correlated with soluble sugar accumulation (McKinley et al., 2016; McKinley et al., 2018; Li et al., 2019). While previous studies have focused on sucrose metabolism enzymes and sugar transporters, the correlation between sugar concentration and enzyme activity has been inconsistent. For example, while sucrose accumulation at anthesis was associated with declining activities of the vacuole invertase and sucrose synthase in stem (Lingle, 1987; McKinley et al., 2016), variation of sucrose concentration among different internodes in sweet sorghum was found to be independent of the activities of sucrose metabolizing enzymes (Lingle, 1987). These observations indicate that sugar transporters may instead contribute to the variation of sucrose concentration in different internodes of sweet sorghum. Sucrose transporters (SUTs) were proposed to be involved in phloem unloading in meristematic zones, as well as in storage parenchyma cells to accumulate sucrose from the stem apoplasmic space of elongating and recently elongated internodes (Milne et al., 2017a; Milne et al., 2017b). More SUTs transcripts were found in the stems of a sweet cultivar compared to a grain one (Milne et al., 2013; Babst et al., 2021). Similarly, *Tonoplast Sugar Transporters* (*TSTs*) are more highly expressed in sweet compared to grain sorghum stems (Bihmidine et al., 2016). Sugars Will Eventually be Exported Transporters (SWEETs), which play a role in phloem loading in leaves (Chen et al., 2012; Xue et al., 2022) and in phloem unloading in sink tissues (Chen et al., 2015; Yang et al., 2018), have also been suggested to be involved in sugar accumulation in sorghum stems (Mizuno et al., 2016).

To seek new candidates responsible for sugar accumulation in addition to the *D* locus, SUTs, and TSTs, we conducted transcriptomic data analysis comparing different genotypes (*dd* vs. *DD*), treatments (decapitation vs. control), and stages post anthesis (10d vs. 3d). We found that starch turnover was activated in stems by head removal, while photosynthesis was suppressed in leaves. Both *SbNAC074A* and *SbbHLH093* were down-regulated upon head removal. We provided genetic evidence to support the role of SbbHLH093 homolog in sugar accumulation in *Arabidopsis*, which strongly suggests its potential to modulate sugar accumulation in sorghum. We also observed that *SbSWEET1A*, active in phloem, was induced by head removal in stems, which indicates SbSWEET1A-mediated hexose flux may contribute to enhancing phloem unloading. Our results suggest that reduced reproductive sink strength and enhanced phloem unloading promote sugar accumulation in sorghum stems, which provides us with new insights into the genetic regulation of sugar accumulation in bioenergy crops.

## Materials and methods

### Plant material and growth conditions

The *D* locus NILs described in a previous study (Xia et al., 2018) were planted in an outdoor field in Urbana, Illinois in the summer of 2019. Heads were removed at the anthesis stage, as defined when the full panicle was out of the flag leaf, and half of the pollen was mature. The control plants were intact. The middle of the second leaf, excluding the flag leaf, and the fourth internode from the top of the plant, were taken as tissue samples. The outer layer of the stem was removed with a razor blade so that only the inner stems were collected at 3- and 10-days post-treatment. Five replicates at each time point for each group per genotype were collected. The *Arabidopsis* mutant *nfl-2* (SALK_121082) was ordered from ABRC. *Arabidopsis* seeds were surface sterilized by 70% ethanol for 5 minutes, rinsed with distilled water three times, and then kept at 4°C for 3-day imbibition. The seeds were sown on ½ Murashige and Skoog (MS) medium supplied with 1% agar. 7-day-old seedlings were transferred to the soil for further growth under 16-h light/8-h dark cycle with 150 µmol m^-2^ s^-1^ light intensity at 22°C.

### Sugar measurement

Stems and leaves were ground into powders in liquid nitrogen. The homogenized samples were incubated in 80% (v/v) ethanol at 80°C for 30 min, then centrifuged at 13,000 rpm for 10 min. The supernatant containing soluble sugars was transferred to a new tube and the solution was evaporated for two days at 42°C. The dry residues were dissolved in deionized water, and the solution was filtered through a 0.45 µm syringe filter. Sugars were separated and quantified by a Rezex RCM-Monosaccharide Ca^+2^ (8%), LC Column. At least three replicates from independent plants were collected.

### Total RNA extraction and mRNA-seq library preparation

Frozen samples were ground into powders in liquid nitrogen with pestles and mortars. Total RNA was extracted using TRIzol (15596018; Invitrogen), and DNA was removed with the Turbo DNA-free Kit (AM1907; Invitrogen). The cDNA libraries were prepared from 1 µg RNA using the KAPA mRNA Hyper Prep Kit (KK8581; Roche) with the KAPA Dual-indexed Adapter Kit for Illumina platforms (KK8722; Roche). The libraries were sequenced with a NovaSeq 6000 (Illumina) at the Roy J. Carver Biotechnology Center at the University of Illinois at Urbana-Champaign.

### Transcriptome analysis

Differentially Expressed Gene (DEG) analysis was performed with the DESeq2 package from Bioconductor in the R environment (Gentleman et al., 2004; Love et al., 2014). Stem and leaf libraries were analyzed separately, and cutoffs were an adjusted *p*-value < 0.01 and a log_2_ fold change > 1 or < -1 under a linear model. The linear model incorporated a term for the treatment, a term for the genotype, and a term for the day, and the coefficients were tested using the usual Wald’s test from the DESeq2 package. Under a linear model, the log_2_ fold change reported by DESeq2 is equivalent to the effect size for that factor. Principal Component Analyses were carried out on the 500 most variable log-transformed gene counts using prcomp from the stats package and ggplot2. Heatmaps were carried out on log-normalized transcript per million (TPM) data converted to z-scores for each gene using the pheatmap (version 1.0.12). There were also heatmaps generated from simple log-transformed TPM values without converting to z-scores. GO analysis was carried out using the Gene Ontology Consortium GO Term Enrichment tools (Mi et al., 2019). Gene Set Enrichment Analysis (GSEA) was carried out using a local version of the GSEA software downloaded from the MSigDB (Subramanian et al., 2005), using gene sets compiled with information on KEGG terms, GO terms, and Pfam designations for each gene collected through the Phytozome database with the BioMart application (Goodstein et al., 2012; Smedley et al., 2015). The genes were analyzed with the pre-ranked GSEA version, and the ranking was performed by the signal-to-noise ratio, which incorporates the log_2_ fold change calculated for each factor effect in DESeq2, and includes the application of the ashr shrinkage estimator correction (Stephens, 2017).

### RNA *in situ* hybridization

The sorghum 4^th^ internode stem samples were embedded in paraffin and the following RNA hybridization process was carried out as previously described (Wang et al., 2021). DNA template for RNA probes was amplified from sorghum cDNA using gene-specific primers (see **Supplementary Table S6**). RNA probes were synthesized *in vitro* using MEGAscript™ T7 Transcription Kit for *SbSWEET1A* probes and MEGAscript™ SP6 Transcription Kit for *SbCIN5* probes (Thermo Fisher Scientific). RNA probes were labeled using fluorescein-12-UTP and were hydrolyzed to ~100 nucleotides. Hybridized probes were detected with Anti-Fluorescein-AP (Sigma-Aldrich) and the substrates 5-bromo-4-chloro-3-indolyl phosphate/nitro blue tetrazolium (BCIP/NBT).

### Statistical analyses and data presentation

Statistical analysis was carried out to identify if differential sugar accumulation could be observed between samples and conditions. After removing outliers and null reads, the sucrose data for each sample was incorporated into a one-way ANOVA for pairwise comparisons, and a three-way ANOVA to identify factor effects. The statistical model used in the three-way ANOVA was as follows:


$$Sugar_{ijk}=Treatment_{i}+\;Genotype_{j}+Day_{k}+Treatment*Day_{ik}+\;\epsilon_{ijk}$$

Where the treatment was contrasting Head Removal and Control, the Day was contrasting 10 days and 3 days post-treatment, and the Genotype was contrasting homozygous *dd* with homozygous *DD* for the *D* Locus NILs. An interaction was incorporated into the ANOVA between treatment and day because the treatment will get more severe with time, leading to greater differences between the decapitated and control plants. Both ANOVAs were carried out separately for the stem tissue and the leaf tissue, and were followed by a Tukey’s Honest Significant Difference using the stats package in R. In both tests, *p-value* < 0.05 was considered a significant difference.

## Results

### Manipulating sugar accumulation in stems of both *DD* and *dd* NILs

**Figure 1:**
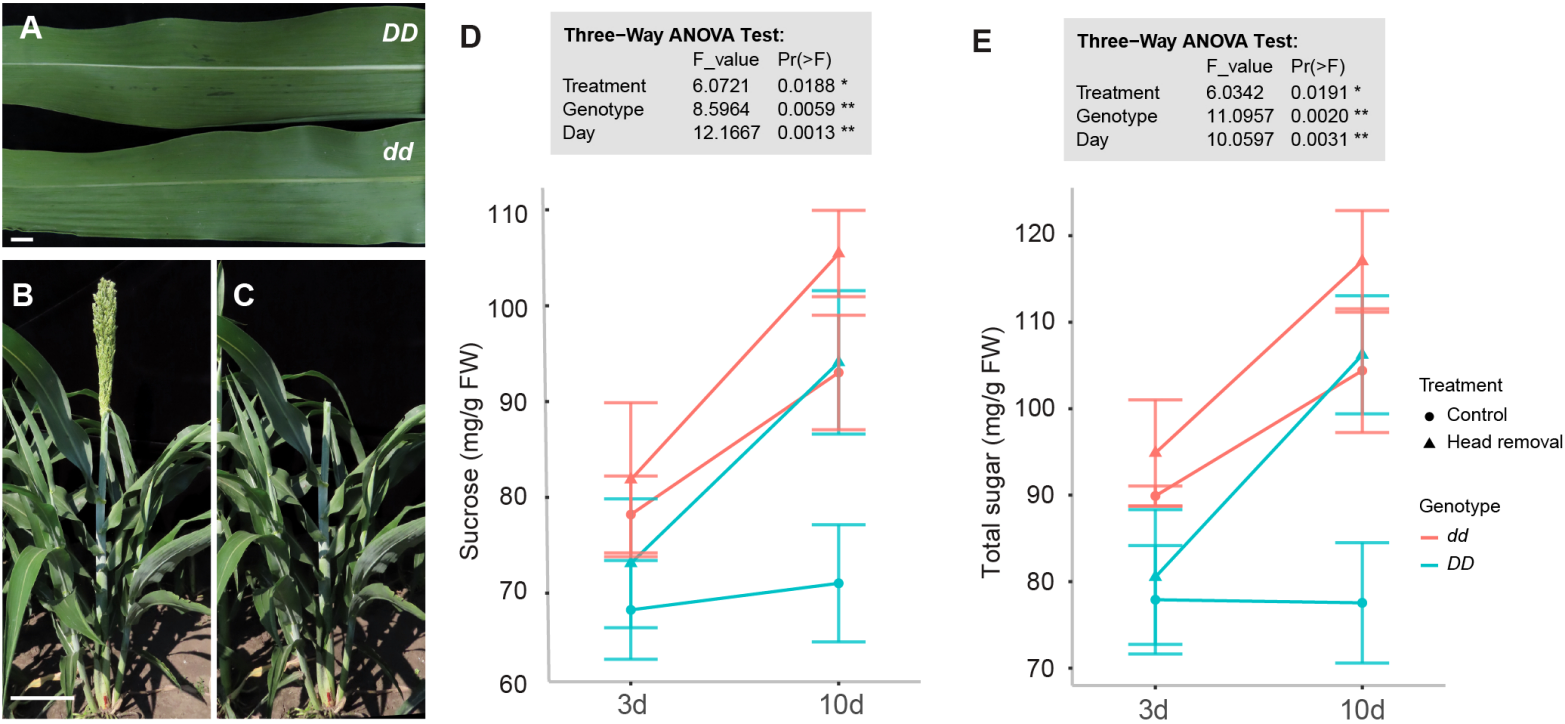
Head removal leads to sucrose accumulation in sorghum stems. **(A)** Leaf midrib phenotype of *DD* and *dd* NILs. **(B, C)** Head removal treatment in the field. Heads were removed at anthesis. **(D, E)** Content of sucrose **(D)** and total sugars **(E)** in stems at 3 and 10 days post head removal (DPHR) in *DD* and *dd* NILs. Values are means ± SE (n = 3 or 4). There are significant differences at *p* < 0.05 in genotype, day, and treatment in three-way ANOVA Tukey’s honestly significant difference test. * *p* < 0.05; ** *p* < 0.01.

*Dry Stalk* (*D*) locus largely determines sugar content and grain yield under well-watered field conditions, and its near-isogenic lines (NILs), juicy green (*dd*) and dry white (*DD*), mainly differ in sugar content with minimal background variability in Tx623 background (Xia et al., 2018). The *dd* NILs with a recessive NAC allele that encodes a truncated transcription factor show green midribs and higher sucrose levels in the stems (Casto et al., 2018; Fujimoto et al., 2018; Xia et al., 2018). The *DD* NILs with a wild-type dominant allele harbor white midribs in fully expanded leaves (**Figure 1A**), and stems have high levels of aerenchyma but low levels of sucrose (Casto et al., 2018; Fujimoto et al., 2018). Although it is in the Tx623 background, *dd* has higher sugar accumulation, imitating the feature of sweet sorghum in terms of sugar content in the stem. Since *DD* and *dd* NILs have almost the same growth characteristics, including flowering time and plant height (Xia et al., 2018), they are ideal materials for studying the contribution of the *D* locus to sugar accumulation in the stem. Therefore, to identify *D* locus-dependent and -independent genes contributing to sugar accumulation, we choose both *DD* and *dd* NILs for further assay.

Soluble sugar in stems increases quickly from 6 days before anthesis to 10 days post anthesis (DPA) in field-grown sorghum plants (McKinley et al., 2016). To isolate more candidates that may play roles in sugar accumulation in addition to *D*, we performed head removal at the anthesis stage (**Figures 1B, C**) since it can further boost the soluble sugar levels in sweet sorghum (Erickson et al., 2012). To focus our investigation on the pith parenchyma cells, which are the primary sugar-storing cells in sorghum stems and minimize the effects of local photosynthesis, we collected the inner tissues of stems without the outer green layer of the stem. First, we measured soluble sugar content at 3 and 10 days post head removal (DPHR) from the fourth internode and the second leaf counted from the top. Sucrose levels in stems were highest in treated *dd* NILs at 10 DPA (**Figure 1D**). The sucrose level differences at *DD* vs. *dd*, 10 DPHR vs. 3 DPHR, and head removal vs. control were statistically significant (three-way ANOVA, p < 0.05), suggesting that sugar in the fourth internode increased in treated plants compared to control plants at either 3 or 10 DPHR in both *DD* and *dd* NILs (**Figure 1E**). In addition, in stems, the levels of glucose and fructose are higher in *dd* compared to *DD*, although they are much lower than sucrose (**Figures S1A, B**). In leaves, no significant difference was detected in either sucrose or total sugar content upon head removal (**Figures S1C, D**). But the levels of glucose and fructose in leaves declined at 10 DPA compared to that at 3 DPA regardless of the treatment or genotype (**Figures S1E, F**). These findings indicate that head removal exerted the expected effects on sugar accumulation in the stem in both genotypes, including the *D*-independent regulations.

### Genes potentially contributing to sugar accumulation by transcriptome analysis

**Figure 2:**
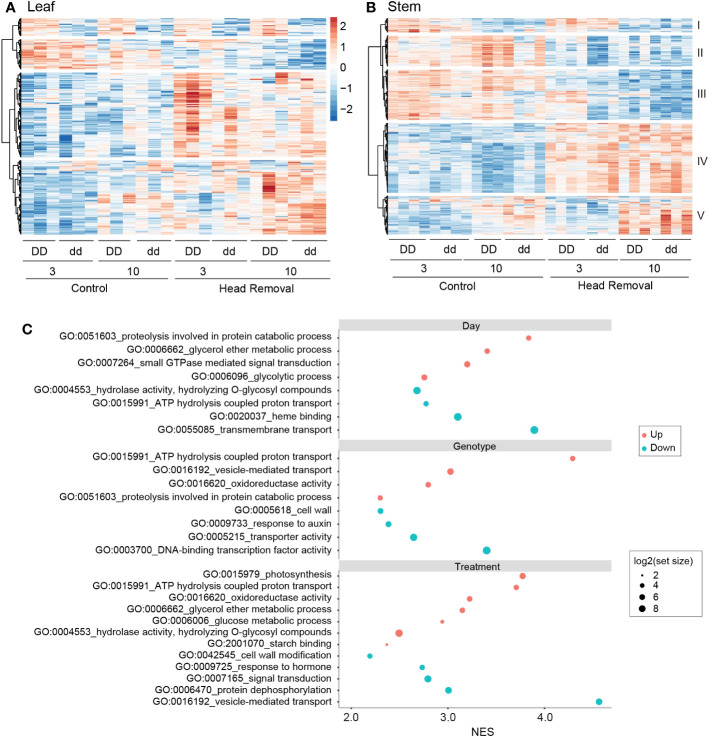
Transcriptome analysis of genes responding to different factors. **(A, B)** Heatmap of differentially expressed genes (DEGs) response to head removal at 3 and 10 DPHR in leaf **(A)** and stem **(B)**. The scale bar shared by **(A, B)** represents the color saturation gradient based on the Log_2_TPM converted to z-scores. Five clusters of DEGs were classified based on co-expression patterns in stems **(B)**. **(C)** Gene Set Enrichment Analysis (GSEA) for stem genes in response to day, genotype and head removal. Gene ontology (GO) terms include biological process, cellular component, and molecular function. Many GO terms are located at multiple levels within the ontology; only the representative ones are displayed here. See the whole GO terms in Dataset S3. The negative normalized enrichment scores (NES) were converted to absolute values shown as blue dots in order to show with positive NES (red dots) on the same side of the y-axis.

To identify genes associated with stem sugar accumulation, we obtained transcriptome profiles from the fourth internode and second leaf of NIL plants. The stem and leaf samples were analyzed separately using the DESeq2 package in R under a linear model (Love et al., 2014). In the coefficient tests, cutoffs of absolute log_2_ fold change ≥ 1 and an adjusted p < 0.01 were implemented to output the differentially expressed genes (DEGs) list. A total of 682 and 1611 DEGs were identified in leaves and stems, respectively. DEGs from each factor (day, genotype, and treatment) were combined to make the hierarchical clustering heatmaps (**Figures 2A, B**; **Datasets S1, S2**). Most of the DEGs in leaves are grouped into two clusters, as shown at the bottom of the heatmap shown in **Figure 2A**, both of which are upregulated by head removal compared to corresponding control plants. Among them, genes response to abiotic stresses, such as hydrogen peroxide, heat and osmotic stress, were enriched (**Table S1**). The stem transcriptome data formed five clusters based on the expression patterns (**Figure 2B**). The DEGs between collection days were grouped into clusters I and V with cluster I showing lower gene expression at 10 DPA compared to 3 DPA, while genes in cluster V were elevated at 10 DPA. Clusters II, III, and IV represented the genes responsive to treatment. Genes in clusters II and III were suppressed by head removal and played roles in ammonium transmembrane transport and auxin-activated signaling pathways. While genes in cluster IV were elevated by head removal and involved in photosynthesis and glycogen biosynthetic process (see the enriched GO terms in **Table S2**).

To examine the effects of each factor on physiological processes with a sensitive approach, we used the same dataset obtained from DESeq2 and conducted gene set enrichment analysis (GSEA), which can generate signal-to-noise ratios as a readout (Subramanian et al., 2005). The day factor in stems showed an upregulation of gene sets involved in protein catabolism along with the treatment and genotype factors. However, it is also associated with a decrease in transmembrane transport and ATP hydrolysis coupled proton transport, which is a departure from both the treatment and genotype factors (**Figure 2C**; **Dataset S3**). The effects of head removal on stems caused the upregulation of many gene sets enriched in functional groups related to carbon metabolism and starch binding, which is consistent with the increased availability of sugar present in the stem. ATP hydrolysis coupled proton transport is upregulated across both the genotype and the treatment, which may increase the strength of the proton gradient and therefore the activity of active sugar transporters without increasing the transcript levels, such as *SbSUT1*. In addition, head removal is associated with a significant but minor increase in pathways related to photosynthesis in stems (**Figure 2C**). In contrast, photosynthesis is downregulated by head removal in leaves (**Figures S2A, S3**). Both genotype and treatment show a decrease of cell wall deposition in stems, implying that more carbon is diverted from the lignocellulose pathway in *dd* NILs and decapitated sorghum. Moreover, there is a downregulation of gene sets involved in auxin responses and signal transduction, such as dephosphorylation and vesicle-mediated transport (**Figure 2C**). These findings indicate that basic biological processes, such as protein synthesis, carbohydrate metabolism and ATP-dependent transport, were enhanced by head removal in stems, but pathways involved in response to endogenous auxin and the external stimuli were inhibited (**Figure 2C**; **Dataset S3**).

### Sugar accumulation is negatively associated with bHLH093 activities

**Figure 3:**
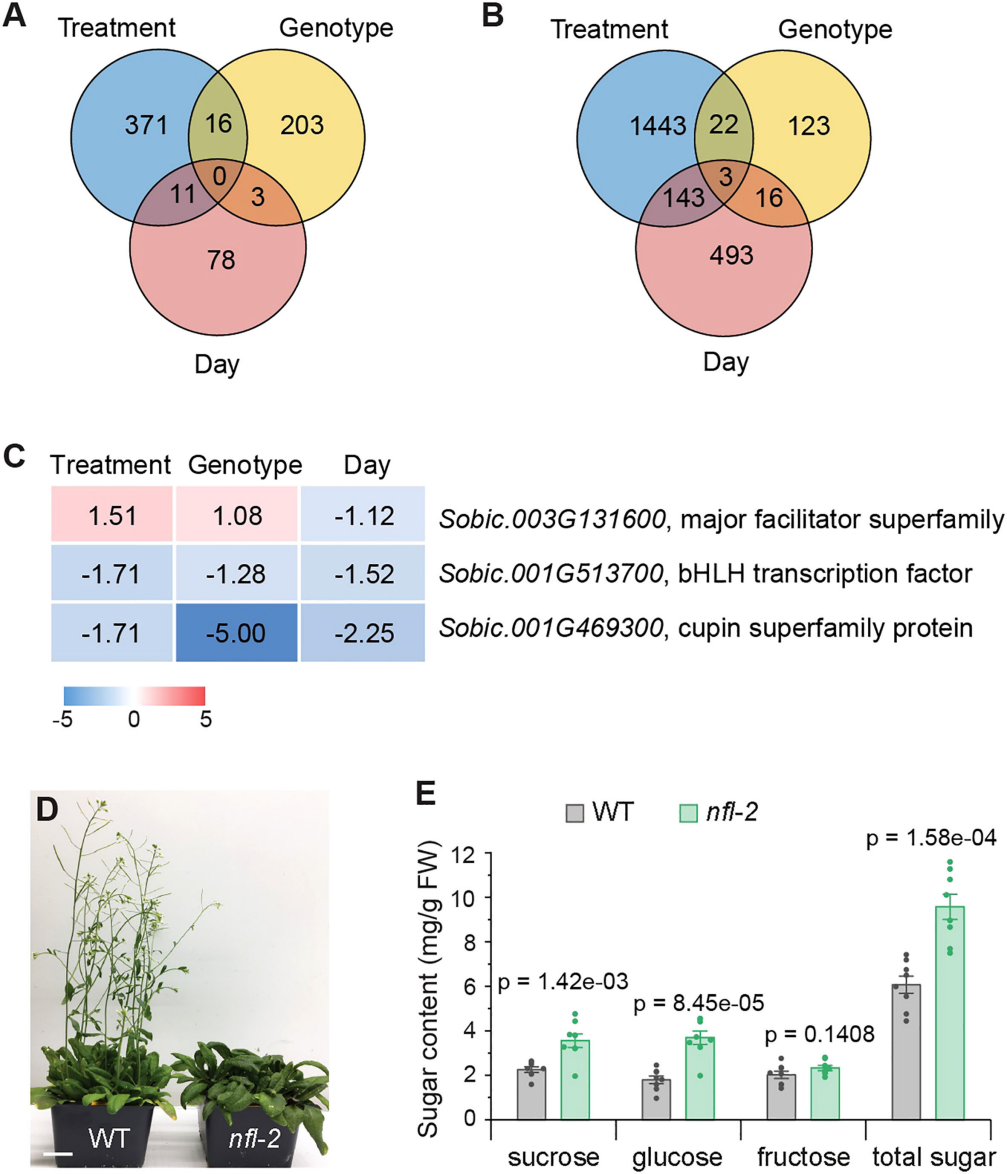
Identification of genes responding to all conditions and functional analysis of a resulting homolog in *Arabidopsis*. **(A, B)** Venn diagram of DEGs response to treatment (decapitation vs. control), genotype (*dd* vs. *DD*), and day (10 DPA vs. 3 DPA) in leaf **(A)** and stem **(B)**. **(C)** Intersection genes of different factors in stem. Color gradient (red-white-blue) applied to a range of Log_2_FC (maximum-minimum). The background color indicates where each cell value falls within that range. **(D)** *nfl-2* shows the later flowering phenotype. Image shows 4-week-old plants. Scale bar = 2 cm. **(E)** Higher levels of sugars detected in *nfl-2*. Sugars in 9^th^ true leaf were extracted from 4-week-old plants. Values are means ± SE (n = 8). Student’s unpaired *t* test is used to calculate two-tailed *p* values when compared with the values in corresponding wild type.

The highest number of differentially regulated genes triggered by treatment in both tissues (**Figures 3A, B**; **Datasets S1, S2**) signify impact of treatment on the overall gene expression patterns. By examining genes responding to all three factors, we aimed to gain better insights into the mechanisms governing sugar accumulation. Interestingly, our findings revealed that no single gene exhibited a response to all factors in leaves (**Figure 3A**), which aligns with the absence of significant sugar elevation induced by either one of these factors. Three genes were found to be regulated by all factors in stems (**Figure 3B**). Among them, the level of *Sobic.003G131600* that encodes a major facilitator superfamily protein, was higher in decapitated sorghum and *dd* NILs, but lower at 10 DPA, compared to the corresponding controls (**Figure 3C**; **Table S3**). *Sobic.001G513700* encodes a homolog of *Arabidopsis* basic helix-loop-helix (bHLH) transcriptional factor AtbHLH093/NFL (NO FLOWERING IN SHORT DAY), which establishes the shoot apical meristem (SAM) during the transition from the vegetative to reproductive growth through gibberellin signaling (Sharma et al., 2016; Poirier et al., 2018). *Sobic.001G469300* encodes a cupin superfamily protein. Both *Sobic.001G513700* and *Sobic.001G469300* were suppressed by all three factors (**Figure 3C**; **Table S3**), which suggests their expression levels may be negatively correlated with sugar accumulation.

A recent study revealed that sorghum *SbbHLH093* is highly expressed in apical dome and intercalary meristems, which is proposed to play a role in the cell cycle and cell division during the internode development (Yu et al., 2022). Loss-of-function mutant of *AtbHLH093* in *Arabidopsis* grown under short day or high light conditions shows late flowering phenotype due to disrupted SAM establishment (**Figure 3D**) (Sharma et al., 2016; Poirier et al., 2018). That implies bHLH093 may have a conserved role in controlling meristem development in the plant kingdom and then impacting sugar accumulation. To evaluate this possibility, we measured sugar contents in *Arabidopsis atbhlh093* null mutant allele *nfl-2* (Sharma et al., 2016), since *atbhlh093* delays flowers and may in turn extend the period for sugar accumulation in vegetative tissues before anthesis. As shown in **Figure 3E**, the levels of sucrose, glucose and total sugars increased in *nfl-2* mutant compared to wild-type plants. This finding implies that downregulation of *SbbHLH093* may alter or affect developmental stages in sorghum, such as SAM establishment and flower timing, which prolongs the photosynthetic period and thereby contributes to the enhanced sugar accumulation in stems. Thus, transition delay from vegetative to reproductive growth could be an additional strategy to increase sugar yield in bioenergy crops.

### Starch turnover is likely accelerated by head removal

It has been reported that starch turnover is higher in sweet sorghum stems (Li et al., 2019). Our data also found that starch metabolism-related genes were substantially altered in decapitated sorghum stems, but not in leaves (**Figure S4**). This finding is further supported by GSEA, where gene sets upregulated after head removal are involved in glycerol ether and glucose metabolism, hydrolase activity of o-glycosyl bonds, as well as an increase in starch binding and the glycogen biosynthetic process, each of which contributes to starch synthesis (**Figure 2C**; **Dataset S3**). Genes encoding isoamylase 1 and starch phosphorylases a and b were induced by head removal in both *DD* and *dd* NILs (**Table 1**). The highest transcript levels and fold changes by induction were observed at 10 DPHR in *dd* NILs for each gene (**Table S4**). Genes encoding isoamylase 3 and alpha-glucan water dikinase were also upregulated by head removal (**Table 1**). The transcript levels of genes encoding each of the ADP-glucose pyrophosphorylase (AGPase) subunits, starch branching enzyme 2.2a and starch synthase 1 increased in decapitated *DD* and *dd* NILs, and show the highest expression levels at 10 DPHR in *dd* NILs (**Table 1**). These results suggest that starch metabolism is promoted by head removal, with higher activity in *dd* NILs, which aligns with the observation that more sugar was accumulated in *dd* NILs (**Figures 1D, E**).

**Table 1:** Effects of treatment, genotype and day on transcript levels of starch and PCD-related genes in the stem.

Gene ID	Gene Name	TPM	Treatment		Genotype		Day	
			Effect size*	*p* value	Effect size*	*p* value	Effect size*	*p* value
Starch metabolism-related genes								
Sobic.007G204600	*Isoamylase 1*	35.58 ± 5.76	1.67	4.77E-15	0.25	0.0752	0.21	0.1649
Sobic.001G083900	*Starch phosphorylase a*	169.06 ± 27.37	1.48	5.72E-10	0.35	0.0256	0.28	0.0841
Sobic.003G358600	*Starch phosphorylase b*	206.50 ± 14.74	0.57	5.03E-04	0.23	0.0689	0.18	0.1883
Sobic.007G101500	*AGPase small subunit a*	90.51 ± 6.17	0.72	1.38E-09	0.39	6.17E-04	0.12	0.2740
Sobic.002G160400	*AGPase small subunit b*	184.20 ± 15.94	1.02	1.27E-15	0.45	2.61E-04	0.25	0.0325
Sobic.001G100000	*AGPase large subunit*	21.71 ± 2.11	1.13	3.24E-14	0.39	3.37E-03	0.04	0.7567
Sobic.002G233600	*Isoamylase 3*	64.28 ± 3.20	0.57	1.03E-09	0.35	1.63E-04	-0.07	0.4566
Sobic.010G143500	*Alpha-glucan dikinase*	75.35 ± 4.78	0.86	8.21E-18	0.08	0.4318	0.18	0.0650
Sobic.010G273800	*starch branching enzyme 2.2a*	229.30 ± 21.55	1.31	4.77E-27	0.21	0.0524	0.35	2.09E-03
Sobic.010G047700	*Starch synthase 1*	211.99 ± 20.17	0.53	1.50E-03	0.98	6.85E-08	0.10	0.4925
Sobic.003G084000	*SbpGlct*	206.88 ± 6.69	0.33	3.74E-06	0.20	8.32E-03	-0.08	0.3527
Sobic.007G065500	*SbpGPT1*	161.96 ± 6.63	-0.31	7.41E-06	-0.27	2.22E-04	0.14	0.0525
Programmed cell death-related genes								
Sobic.006G147400	*SbNAC074A*	11.72 ± 2.49	-0.73	0.0163	-2.94E-03	0.9838	-2.90	3.51E-12
Sobic.008G020700	*SbMIF*	211.25 ± 18.71	-1.03	1.70E-08	-0.04	0.74189	-0.02	0.9239
Sobic.007G172100	*SbXCP1*	13.30 ± 4.60	-0.09	0.6589	-6.88	1.48E-21	-0.11	0.4900
Sobic.003G087200	*BFN1 homolog*	147.46 ± 14.07	-0.59	4.03E-03	0.13	0.3118	-0.17	0.2566
Sobic.004G187200	*Type II metacaspase*	2.07 ± 0.41	-0.63	0.0364	-0.07	0.5466	0.14	0.4011
Sobic.004G010000	*SCPL48 homolog*	2.97 ± 0.59	-0.61	0.0363	-0.34	0.0311	-0.27	0.1505

*Estimated effect size under a linear model in DESeq2 is equivalent to the log_2_FC in a pairwise comparison.

Color gradient (red-white-blue) applied to a range of effect size values (maximum-minimum). The background color indicates where each cell value falls within that range.

TPM, transcript per million. The TPM value is mean ± SE (n = 29).

Transcript levels of each gene under different conditions in stems are shown in **Table S4**.

Another piece of evidence to support starch metabolism being changed by head removal are the levels of plastidic sugar transporters. A plastidic glucose translocator (pGlcT) gene (*Sobic.003G084000*) was induced at 3 DPHR at *DD* and *dd* NILs, indicating that more products degraded from starch were exported out of plastids (**Figure S5A**; **Table 1**). Glucose-6-phosphate/phosphate translocators (GPTs) play roles in sugar sensing (Hanson and Smeekens, 2009). Plants with impaired starch biosynthesis had higher expression of *AtGPT2* (Kunz et al., 2010). In decapitated sorghum, the *SbGPT1* (*Sobic.007G065500*) transcript level was lower in stems, implying that starch metabolism activities are higher (**Figure S5B**; **Table 1**).

### Head removal delays the expression of genes associated with PCD in stems

It has been reported that the *D* locus gene *SbNAC074A*, negatively associated with sugar accumulation in sorghum stems, positively regulates programmed cell death (PCD) to form aerenchyma (Casto et al., 2018; Fujimoto et al., 2018; Xia et al., 2018). We therefore investigated whether the further sugar accumulation by head removal was attributed to *SbNAC074A* and its associated genes such as the miniature zinc finger (*SbMIF*) gene (Xia et al., 2018). The transcript level of *SbNAC074A* was suppressed by head removal in *DD* NILs at 3 and 10 DPHR (**Table 1**). Although the SbNAC074A protein produced in *dd* NILs is truncated and nonfunctional (Xia et al., 2018), the transcript level of *SbNAC074A* was still down-regulated by head removal at 10 DPHR, indicating that a shared upstream signal triggered by head removal boosts the juice volume by suppressing *SbNAC074A*, probably involving sugar signaling and/or auxin signaling as aforementioned. The expression of *SbMIF*, encoding a truncated zinc finger-homeodomain transcriptional factor (ZF‐HD) and a suspected target of SbNAC074A (Xia et al., 2018), was highly reduced at 3 and 10 DPHR in *DD* and *dd* NILs after head removal (**Table 1**; **Table S4**). This indicates that although *MIF* and *Dry* share a similar expression pattern, this regulation may occur through parallel or redundant pathways regardless of the presence or absence of functional SbNAC074A.

Fujimoto et al. (2018) found that SbNAC074A induces cell death by activating the expression of the downstream PCD-executing enzymes. Therefore, we checked whether genes encoding PCD-executing enzymes were suppressed by head removal as well in stems. The transcript of *SbXCP1*, encoding a cysteine protease, was downregulated by head removal in *DD* NILs but was barely detected in *dd* NILs (**Table 1**; **Table S4**). This finding is consistent with *SbXCP1* expression being dependent on a functional SbNAC074A (Casto et al., 2018). In addition, a BFN1 homolog *Sobic.003G087200*, a type II metacaspase homolog *Sobic.004G187200*, and an SCPL48 homolog *Sobic.004G010000* were suppressed by head removal in both *DD* and *dd* NILs (**Table 1**; **Table S4**). These data suggest that head removal delays the PCD in stem pith in a SbNAC074A-dependent and -independent manner, resulting in a prolonged duration of stem development, and in turn an increase in juice volume and sugar yield. This finding also explains why the net increase of sucrose is higher in *DD* NILs, which may be attributable to a *D* locus-dependent PCD pathway (**Figure 1D**).

### Sugar transporters contribute to sugar accumulation in the stems

**Figure 4:**
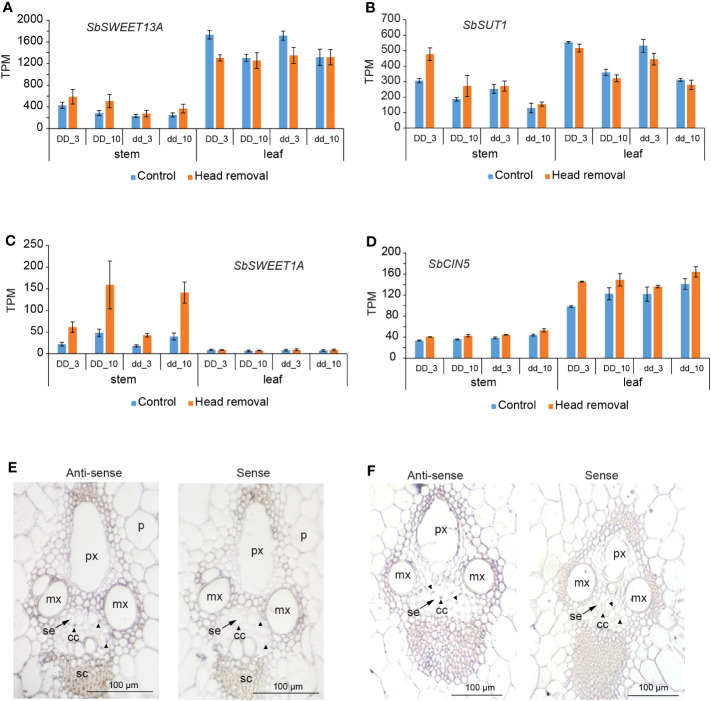
Transcript profiles of sugar transporter genes upon head removal. **(A, B)** Transcript levels of *SbSWEET13A* **(A)** and SbSUT1 **(B)** upon head removal in stem and leaf. **(C)** *SbSWEET1A* was induced by head removal in stems. **(D)** *SbCIN5* encoding a cytosolic invertase was slightly induced by head removal in stem and leaf in all tested conditions. Values are means ± SE (n = 3 or 4). Effect sizes and *p* values are shown in **Table S5**. **(E, F)** Spatial expression analysis of *SbSWEET1A* **(E)** and *SbCIN5* **(F)** by RNA *in situ* hybridization in the 4^th^ internode at 10 DPHR in *dd* NILs. Sections hybridized with the sense probes serves as background control. p, pith parenchyma; mx, metaxylem; px, protoxylem; se, sieve elements (arrows); cc, companion cells (arrowheads); sc, sclerenchyma cells.

Increased sucrose accumulation in stems upon head removal must result from at least one of these changes: increased photosynthetic rate, enhanced phloem loading in source leaves, elevated phloem unloading, or strengthened storage in stem vacuoles. However, it cannot be simply explained by the removal of competitive reproductive tissue because the relationship between stem sucrose content and grain yield is not a simple physiological trade-off (Gutjahr et al., 2013). The transcriptome profile uncovered that the photosynthesis pathway was suppressed in leaves upon head removal (**Figure S2A**). SbSWEET13A and SbSUT1 have been indicated to play a critical role in phloem loading in sorghum (Milne et al., 2013; Mizuno et al., 2016; Milne et al., 2017b; Chen et al., 2022). *SbSWEET13A* is most highly expressed within clade III SWEET members in sorghum stems and leaves (Mizuno et al., 2016). Maize homologs ZmSWEET13a, b and c active in bundle sheath and phloem parenchyma cells (Bezrutczyk et al., 2018; Bezrutczyk et al., 2021) and ZmSUT1 active in sieve element-companion cell complex (SE-CC) likely function in phloem loading (Baker et al., 2016; Babst et al., 2022). Thus, we first investigated the transcript levels of *SbSWEET13A* and *SbSUT1* in leaves upon head removal. *SbSWEET13A* decreased slightly in leaves upon treatment at 3 DPHR in both *DD* and *dd* NILs (**Figure 4A**). No significant changes in *SbSUT1* transcript levels were detected upon treatment (**Figure 4B**). These data suggest that phloem loading in decapitated plants is comparable or even weakened compared to that in control plants. Therefore, it is unlikely that the increased sucrose levels in stems caused by head removal are due to increased sugar production in source leaves or increased phloem loading. These are consistent with an overall reduction in sink demand due to the removal of the active sink tissue, which in turn leads to feedback downregulation of photosynthesis (Paul and Driscoll, 1997; Paul and Pellny, 2003). Consequently, the total sugar content of leaves is unaltered (**Figure S1C**) despite the reduction in photosynthesis (**Figure S2A**).

SWEETs have been reported to function in sugar flux in sink tissues as well (Chen et al., 2015). We investigated the transcript levels of all 23 *SbSWEET* genes in stems upon treatment and found that *SbSWEET1A* was induced by head removal at 3 and 10 DPHR in both *DD* and *dd* NILs (**Figure 4C**). Correspondingly, the transcript level of *SbCIN5* (*Sobic.004G163800*), which encodes a cytoplasmic invertase, also slightly increased after head removal (**Figure 4D**). RNA *in situ* data showed that transcript of *SbSWEET1A* and *SbCIN5* were detected in companion cells, sclerenchyma cells, as well as pith parenchyma cells (**Figures 4E, F**). These data imply that glucose flow from vascular cells to the storage pith cells is elevated (**Figure 4E**). Upregulation of *SbSWEET1A* and *SbCIN5* may help move more hexose from the phloem to the apoplasmic space, increasing hydrostatic pressure differences in the phloem when a strong sink is removed, which is beneficial for the bulk flow from source leaves to sugar-accumulating stems. This aligns with the finding that none of the clade III members are upregulated by head removal except for *SbSWEET13A*, which is slightly induced (**Figure 4A**). We found *SbSWEET1A* is also expressed in pith parenchyma cells (**Figure 4E**), implying that SbSWEET1A may take up glucose from the apoplasmic space too.

Sucrose in apoplasm is taken up by storage cells through SUTs (Milne et al., 2013; Milne et al., 2017a) and then pumped into vacuoles by TSTs (Bihmidine et al., 2016). The sugar in the vacuole may be exported to the cytosol by tonoplast-localized SUTs (Endler et al., 2006; Eom et al., 2011) or by clade IV SWEET members (Klemens et al., 2013). Increased tonoplast-localized AtSWEET16 led to less sugar accumulation at the end of the night (Klemens et al., 2013). Interestingly, we found *SbSWEET16* transcript levels decreased in decapitated plants (**Figure S6A**), which may lower sugar export from the vacuole, facilitating sugar storage there and likely contributing to sugar accumulation in stems. Therefore, increased sucrose accumulation in stems likely results from elevated phloem unloading accompanied by enhanced vacuole storage. Our findings support the conclusion proposed by Babst et al. (2021), where sugar accumulation in sweet sorghum is driven by elevated phloem unloading and storage in mature stem internodes rather than by increased photoassimilate export in leaves (Babst et al., 2021).

## Discussion

### Source-sink perturbation is a valuable way to study the source-sink relationship

The manipulation of the source-sink relationship can be achieved in several ways, including reducing CO_2_ fixation in the source leaves by defoliation or leaf shading, or limiting competitive sink tissues by removal of the pod or the whole reproductive tissue. However, defoliation and leaf shading can result in sugar reduction in stems (McCormick et al., 2008), so we chose to use head removal that results in sugar increase in stems (**Figure 1**), which facilitates our understanding of the mechanism underlying sugar accumulation in sorghum stems. Although head removal triggered wounding responses, we sampled leaves and stems at 3 and 10 DPHR to minimize side effects caused by mechanical injury. In addition, we also made parallel comparisons at different days post anthesis in different genotypes without treatment. With these efforts, we did not see wounding-responsive genes significantly changed by head removal (**Figure 2C**; **Dataset S3**). In addition to decapitated sorghum, male sterile lines are another option to eliminate grain sinks. Recently, Jebril et al. found that sterile sweet sorghum hybrids produce more sugar in stems than fertile ones (Jebril et al., 2021). Therefore, male-sterile lines can serve as alternatives to investigate the source-sink relationship without wounding, as long as the sterile lines and the fertile control plants have the same genetic background.

### Delayed PCD contributes to increased sugar contents in stems

Panicles and stems are the major sink tissues importing the photoassimilates after anthesis in grain and sweet sorghum, respectively (Babst et al., 2021). There are no increased photoassimilates produced in source leaves (**Figure S1**), while the sucrose content in stems increases, likely as a result of removal of the competitive panicles. The stem pith and parenchyma cells may passively receive excess sucrose present in the transport phloem. Elucidating the underlying mechanism of stems attracting more photoassimilates under certain conditions will help us to identify more components involved in phloem unloading and thereby better understand the sugar allocation in sorghum. Certainly, it is impossible to exclude the other possibility that head removal alters other pathways to actively enhance phloem unloading and storage capacity in stems, such as auxin signaling-activated cell division and elongation to promote internode growth, resulting in a prolonged duration of stem development.

The generation of *dd* and *DD* NILs allowed us to discover both *D*-dependent and -independent pathways for increasing sugar content upon head removal. Our data supported that increased sugar accumulation caused by head removal resulted from both *D*-dependent and -independent pathways. In addition, SbXCP1-mediated PCD contributing to sugar accumulation is indicated downstream of a functional SbNAC074A (Casto et al., 2018). Most of the sweet sorghum cultivars harbor loss-of-function alleles of the *D* locus, and it is therefore proposed that a *D* locus mutation was a major target during sweet sorghum selection (Zhang et al., 2018). These *D*-independent pathways have greater potential to serve as strategies for sugar accumulation improvement in the sweet sorghum background in the future.

### Delayed transitioning to reproductive development contributes to sugar accumulation in stems

In this study, we found some *D*-independent regulators, including SbbHLH093. *SbbHLH093* was expressed at the highest level in the apical dome and its expression was gradually lowered towards the mature phytomer (Yu et al., 2022). These data indicate the *SbbHLH093* is likely to be expressed at a substantially high level in the SAM of sorghum plants. This expression pattern aligns with the function of its Arabidopsis homolog, AtbHLH093/NFL (NO FLOWERING IN SHORT DAY), which has been demonstrated to play a critical role in establishing the SAM during the transition from the vegetative to reproductive growth through gibberellin signaling (Sharma et al., 2016; Poirier et al., 2018). Soluble sugar in Della sorghum stems started to increase after floral induction (~ 49 days before anthesis) and reached to peak at 26 days post anthesis (McKinley et al., 2016). The fastest accumulation was from 6 days before anthesis to 10 days post anthesis in Della sorghum stems (McKinley et al., 2016). Given these observations and findings, we proposed that down-regulation of *SbbHLH093* in sorghum stems could lead to delayed transition from vegetative to reproductive phase, resulting in delayed anthesis in sorghum. Therefore, the sugar accumulation period before anthesis is extended, leading to more soluble sugar accumulation in stems.

Anthesis delaying caused by down-regulated *SbbHLH093* may be due to delayed establishment of SAM since *SbbHLH093* is expressed at the highest level in the apical dome (Yu et al., 2022). Of course, we cannot exclude the impact on intercalary meristems (Yu et al., 2022). As intercalary meristems are involved in stem elongation, any delay in their development could result in SAM establishment delay. Consequently, it could lead to late anthesis and higher sugar accumulation in the stems. The findings that *SbbHLH093* downregulation accompanies increased sugar upon head removal in both *dd* and *DD* (**Figure 3C**; **Table S3**), and the loss of *bHLH093* in *Arabidopsis* leads to sugar increase in addition to late flowering (**Figures 3D, E**) support the notion that enhanced sugar accumulation resulting from the disrupted bHLH093. It is known that the volume of stem storage cells available for sucrose storage is determined by the internode length and number, as well as the onset of aerenchyma formation that coincides with cessation of sucrose accumulation (Hilley et al., 2017; Casto et al., 2018). Further investigation is needed to determine whether *SbbHLH093* down-regulation leads to increased internode length and/or number and delayed aerenchyma formation. Regardless of the aforementioned scenarios, a delay in flowering could lead to an extended period of sugar accumulation and/or enhanced storage capacity, which in turn could enable more sugar accumulation in sorghum stems before allocation to other sink tissues. Such a strategy could potentially be applied to other bioenergy crops for boosting sugar yield.

### Sugar transporters synergistically boost sugar contents

The sugar yield is boosted likely due to the reduced function of SbNAC074A and enhanced phloem unloading, as mentioned above. Sorghum employs apoplasmic sucrose phloem loading strategy, as supported by dye tracer methods (Bihmidine et al., 2015; Milne et al., 2015). However, contrasting results were observed for sucrose phloem unloading from the different sorghum species. For instance, applying the apoplasmic tracer, 8-hydroxypyrene-1,3,6-trisulfonic acid, at anthesis via the transpiration stream resulted in its confined accumulation within the innermost boundary of the sclerenchyma sheath cells of a maturing sorghum internode, indicative of radical apoplasmic discontinuity between vascular bundles and storage parenchyma cells, which aligns with symplasmic unloading (Milne et al., 2015). Conversely, introducing the membrane impermeable phloem mobile dye, carboxyfluorescein, into mature source leaves of intact sorghum at anthesis led to its restriction to the phloem of mature stem tissue in both grain and sweet sorghum cultivars (Bihmidine et al., 2015). This suggests a symplasmic discontinuity between sieve element/companion cell complexes and adjacent cells of mature internodes, consistent with an apoplasmic unloading (Milne et al., 2015). These divergent findings could result from differences, such as in analyzed cultivars, sampled internodes and developmental stages. Nonetheless, it is possible that a transition from an apoplasmic to symplamic phloem unloading pathway occurs as the internode matures. Nevertheless, the presence of apoplasmic phloem unloading for mature sorghum internode cannot be completely ruled out. This could occur between sieve element/companion cell complex and phloem parenchyma before sugars reach the lignified and suberized sclerenchyma sheath cells, which presumably hinder water and solute movement; this could potentially occur after sugars bypass the apoplasmic barrier via a symplasmic pathway. In addition, the formation suberized and lignified cell walls may not serve as an absolute barrier to completely prevent solute transport (Bihmidine et al., 2015; Milne et al., 2017a). In light of these complexities, sugar transporters are likely required to play a critical role in facilitating the apoplasmic transfer of sucrose or its hexose derivatives to stem storage parenchyma, aligning with the findings from various studies on the potential roles of sugar transporters in phloem unloading and sugar storage in sorghum (Milne et al., 2013; Bihmidine et al., 2016; Mizuno et al., 2016; Milne et al., 2017a).

Considering our sampling of internode 4, counted from the top after anthesis, which was considerably shorter than fully elongated internode, it likely consists of a substantial portion of enriched meristematic, elongating and recently elongated zones (Milne et al., 2015). These zones predominantly employ apoplasmic unloading, or a combination of symplasmic and apoplasmic transport, consistent with the detected transcripts of the sugar transporters (**Figure 4**; **Table S5**). Plasma membrane-localized sugar transporter SWEETs have been proposed to mediate sucrose efflux from the phloem to the apoplasmic space (Mizuno et al., 2016). In the current study, the transcript levels of S*bSWEET3A* at 3 DPHR in *DD*, and *SbSWEET4A* at 3 and 10 DPHR in *DD* and *dd* decreased after head removal (**Figures S6B, C**). Although it is unclear how and where SbSWEET3A and SbSWEET4A function, especially in response to head removal, the transcript changes are consistent with the findings that the expression of *SbSWEET3A* and *SbSWEET4A* are relatively lower in sweet sorghum internodes compared to that in grain sorghum (Li et al., 2019). These data suggest that the reduction of *SbSWEET3A* and *SbSWEET4A* is also associated with elevated sugar in stems.

*SbSWEET1A* and *SbCIN5* transcripts were detected in all vascular tissues, including phloem companion cells, and were upregulated by head removal (**Figures 4C–F**), indicating increased hexose efflux from the phloem to the vascular apoplasmic space. Given that *SbSWEET1A* was induced more than two times, and considering that two molars of hexoses can result from one molar of sucrose through invertase cleavage, a reduction in sugar concentration within the companion cells of the unloading site likely occurs, contributing to altered osmotic pressure (Milne et al., 2018). Thus, the upregulated *SbSWEET1A* potentially aids in lowering sugar concentration at the unloading site in order to maintain or enhance hydrostatic pressure differences between the loading and unloading sites within the phloem, which could subsequently impact mass flow rate and/or minimize any feedback effects on phloem loading. However, we currently lack substantial evidence supporting the phloem flow rate maintenance or a quantitative model illustrating the impact of hexose efflux on sucrose unloading, but this presents an intriguing mechanism that is worth further exploration. *SbSWEET1A* is also expressed in the pith parenchyma cells, indicating its potential involvement in hexose uptake from the apoplasm for storage or adjustment of cell turgor together with other plasma-membrane localized and tonoplast-localized sugar transporters, such as SUTs and TSTs (Bihmidine et al., 2016; Milne et al., 2017a). The genetic evidence regarding the specific phloem loading, unloading strategy employed and transporter-involved sugar storage in sorghum remains limited. Therefore, there is an urgent need for future investigations aimed at enhancing our understanding of sugar transport mechanisms in sorghum.

Our data further support that SUTs, especially SbSUT1 (**Figure 4B**), play a critical role in accumulating sucrose from the apoplasmic space of recently elongated internodes (Milne et al., 2017a). *Sobic.003G131600*, which encodes a tonoplast-localized major facilitator superfamily protein, was higher in decapitated sorghum and *dd* NILs (**Figure 3C**). Although the activities of Sobic.003G131600 are unknown, we could not exclude the possibility that Sobic.003G131600 transports sugar or a Zn-ligand complex into vacuoles, which contributes to sugar accumulation in sorghum stem. Further studies are needed to address how sugar acts as a signaling molecule to regulate the expression of these sugar transporter genes at the transcriptional level.

## Conclusions

Overall, we found more regulators and sugar transporters that are involved in sugar allocation and storage using source-sink manipulation as a valuable study tool, providing new insights into how to improve sugar accumulation for biofuel production. Downregulation or knocking out *SbbHLH093* may further boost sugar content in sweet sorghum. Further study is needed on the extent to which sugar levels can be increased when *SbSWEET1A* is overexpressed in sorghum or other biofuel crops. In addition, this study implies additional strategies for improving sugar accumulation in stems by genetically limiting reproductive tissues, such as by impairing panicle development. Stacking these strategies may be considered for a further enhancement of sugar accumulation.

## Data availability statement

The RNA-sequencing data described in this study have been deposited in the National Center for Biotechnology Information GEO database, can be accessed with accession no. GSE206420.

## Author contributions

XX and L-QC planned and designed the experiments. XX, GB, JW and Y-CY conducted the experiments and XX, GB and L-QC analyzed the data. SM grew *DD* and *dd* NILs plants in the field. JC guided RNA-seq data analysis. XX, GB and L-QC wrote the manuscript. All authors reviewed the manuscript. All authors contributed to the article and approved the submitted version.
