# Supplementary material for: Sugar accumulation enhancement in sorghum stem is associated with reduced reproductive sink strength and increased phloem unloading activity
Source: Front Plant Sci. 2023 Sep 11;14:1233813. doi: 10.3389/fpls.2023.1233813 (PMC10519796; doi:10.3389/fpls.2023.1233813)
Supplement: Supplementary file 4 [file DataSheet_4.docx]

Supplementary Material

**Sugar accumulation enhancement in sorghum stem is associated with reduced reproductive sink strength and increased phloem unloading activity**

Xueyi Xue, Gabriel Beuchat, Jiang Wang, Ya-Chi Yu, Stephen Moose, Jin Chen and Li-Qing Chen^†^

^†^**Correspondence**: Corresponding Author: [lqchen77@illinois.edu](mailto:lqchen77@illinois.edu)

# Supplementary Figures and Tables

## Supplementary Figures


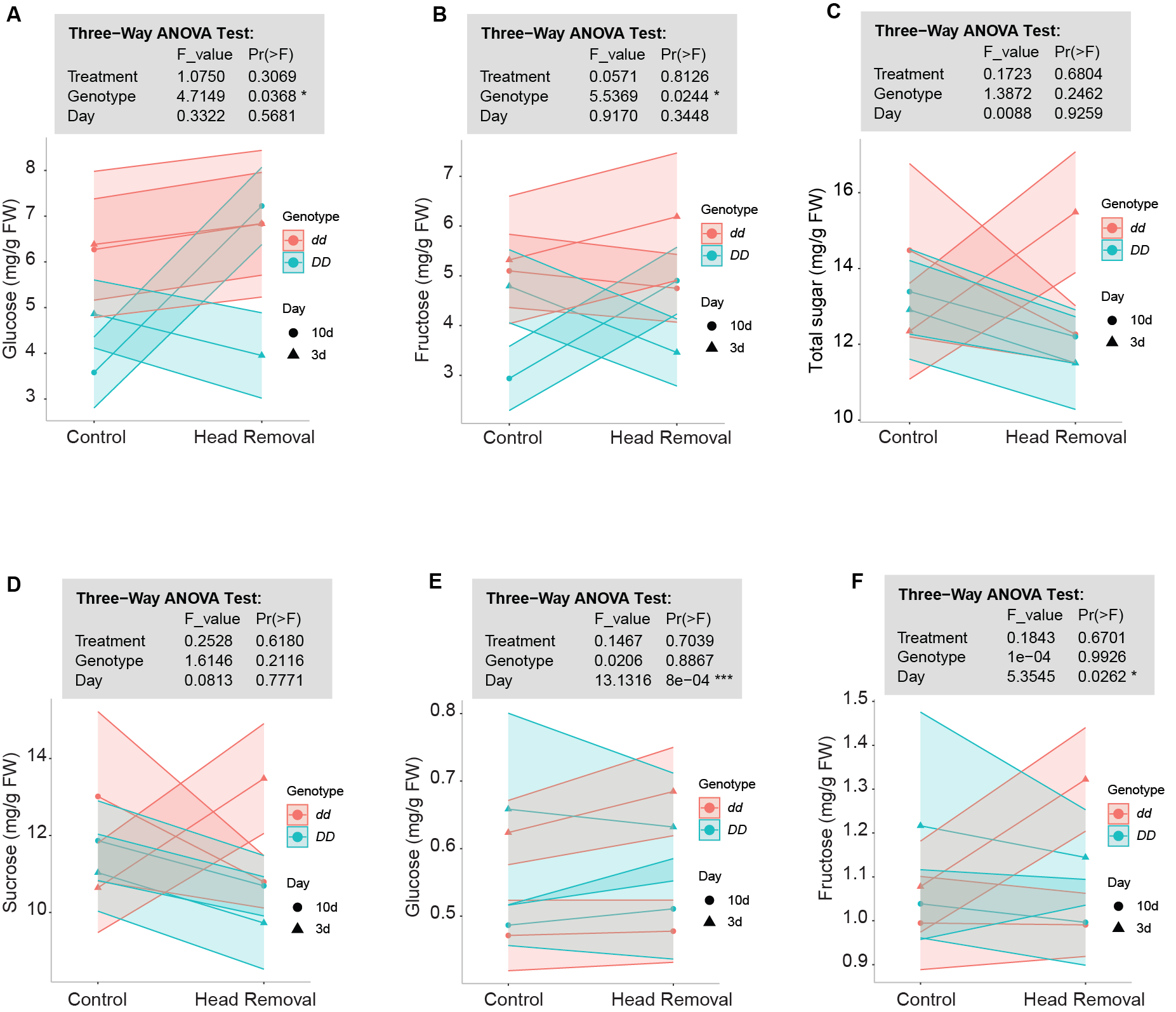


**Supplementary Figure 1. Sugar contents after head removal.** (A, B) Content of glucose (A) and fructose (B) in stems at 3 and 10 DPHR in *DD* and *dd*. There is no significant difference between either day or treatment in both *DD* and *dd* NILs. (C-F) Sugar contents in leaves after head removal. There is no difference in term of levels of total sugars (C) and sucrose (D) in leaves. While the levels of glucose (E) and fructose (F) decreased at 10 DPA in leaves. Values are means ± SE (n = 3 or 4).


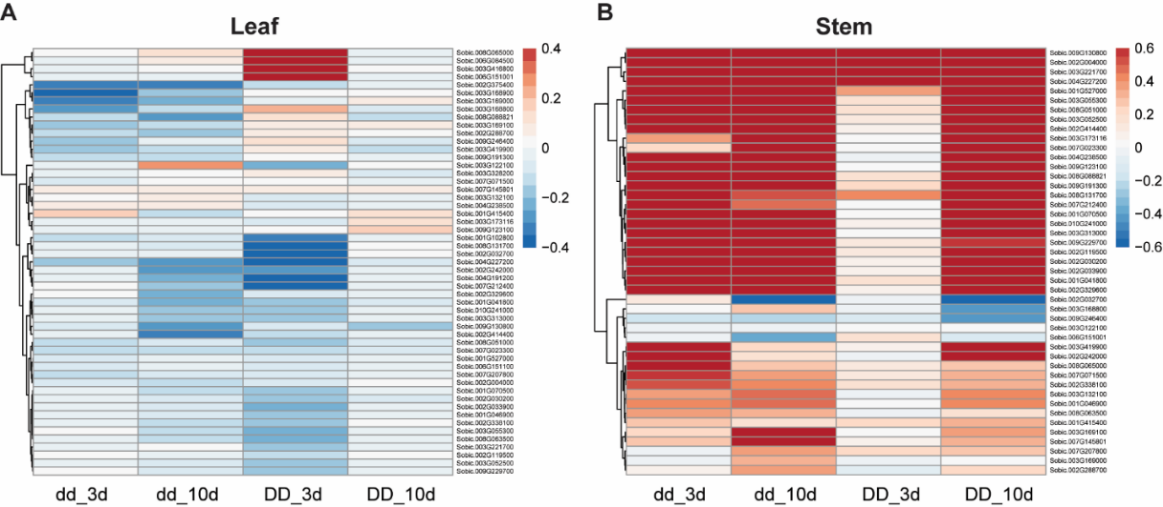


**Supplementary Figure 2. Expression profiles of photosynthesis genes upon head removal.** (A-B) Heatmap of photosynthesis genes in leaf (A) and stem (B) upon treatment. The relative changes of gene expression are presented as log_2_ fold change (log_2_FC) compared to the corresponding controls.


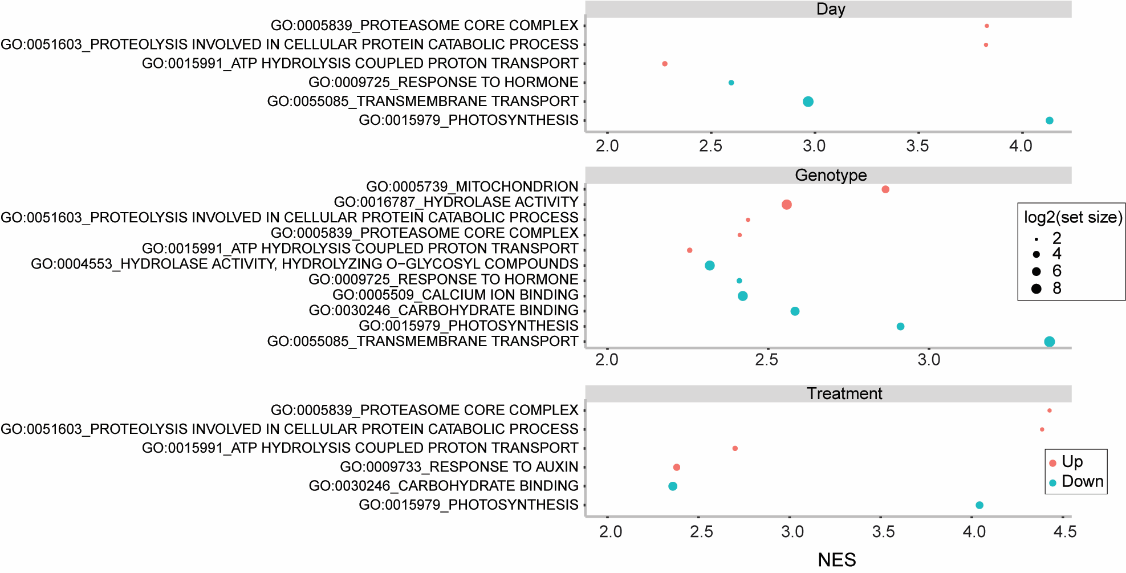


**Supplementary Figure 3.** **GSEA for leave gene expression in response to treatment period, genotype and head removal.** Only the representative ones were displayed. The negative NES were converted to absolute values shown as blue dots in order to show with positive NES (red dots) on the same side of the y-axis. See the whole GO terms in Dataset S3.


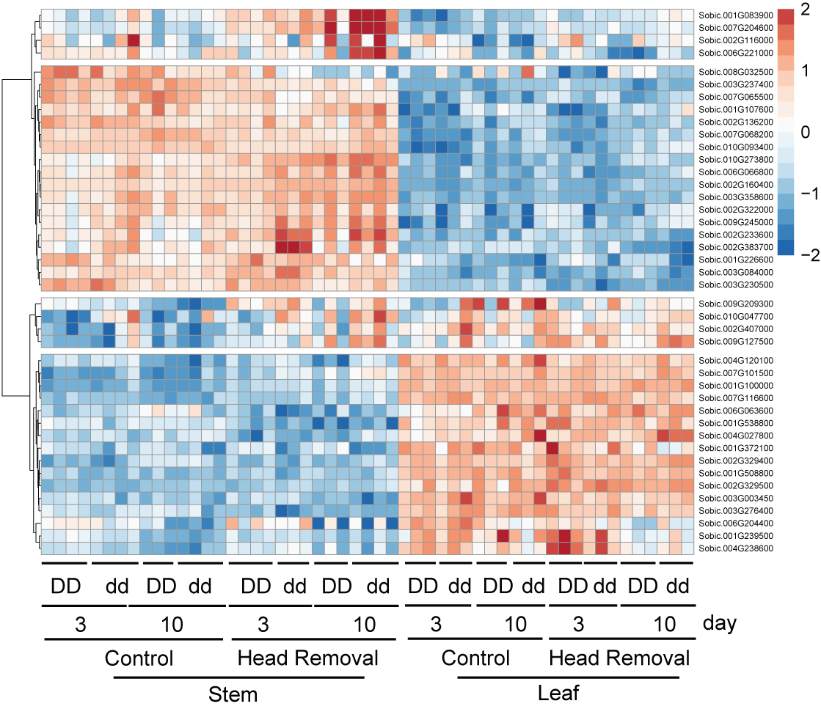


**Supplementary Figure 4. Starch metabolism-related genes were upregulated by head removal in stems.** (A) Heat map of starch metabolism-related genes in stem and leaf upon treatment. The scale bar represents color saturation gradient based on relative transcript expression with a z-score. Effect sizes and *p* values of key genes functioning in starch metabolism are shown in Table 1.


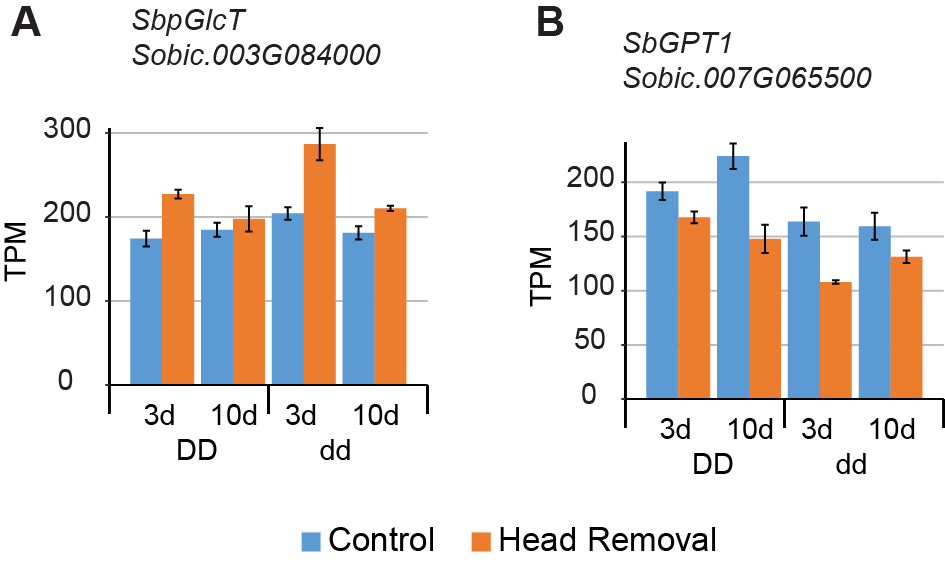


**Supplementary Figure 5. Transcript levels of *SbGlcT* and *SbGPT1* upon head removal.** (A) *SbGlcT* increased after head removal in stem. The induction degree is higher at 3 DPHR in both *DD* and *dd*. (B) *SbGPT1* decreased in stems after head removal in all tested conditions. Values are means ± SE (n = 3 or 4). Effect sizes and *p* values are shown in Table 1.

**
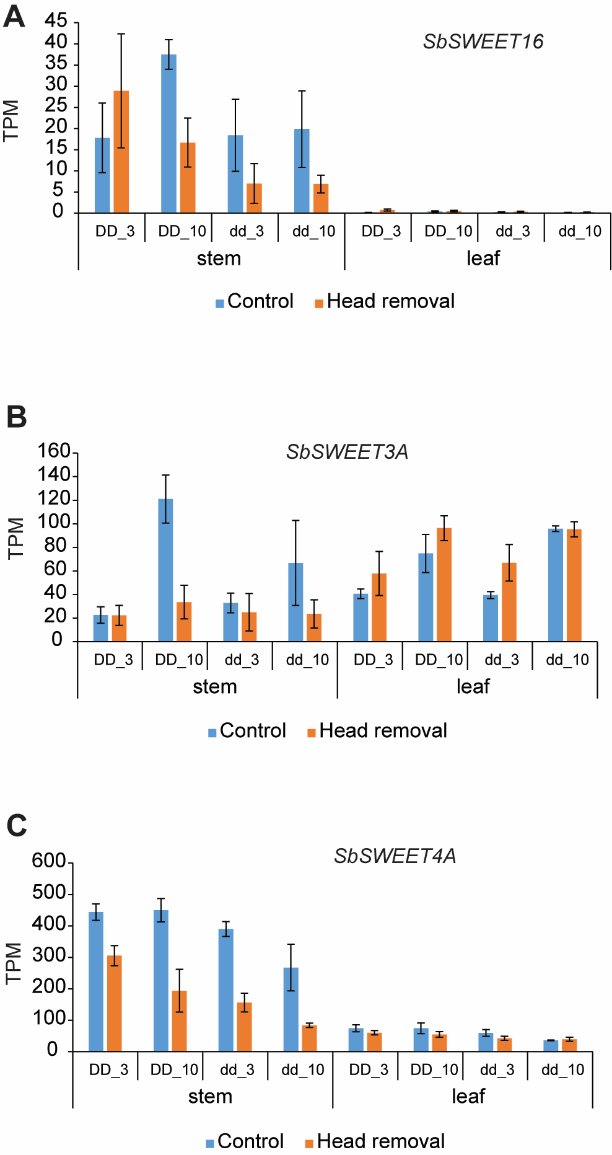
**

**Supplementary Figure 6. Transcript levels of *SbSWEET16*, *SbSWEET3A* and *SbSWEET4A* upon head removal.** (A) *SbSWEET16* was suppressed by head removal in stem. *SbSWEET16* transcripts were barely detected in leaves. (B) *SbSWEET3A* was suppressed in stems by head removal at 10 DPHR. (C) *SbSWEET4A* decreased in stems after head removal at 3 and 10 DPHR. *SbSWEET4A* levels were relatively lower in leaves. Values are means ± SE (n = 3 or 4). Effect sizes and *p* values are shown in Table S5.

## Supplementary Tables

**Supplementary Table 1**. GO term enrichment of leaf DEGs

GO term enrichment of clusters III and IV from top in Figure 2A

| **GO biological process complete** | **Sorghum bicolor - REFLIST (33714)** | **upload_1 (258)** | **upload_1 (expected)** | **upload_1**  **(over/und er)** | **upload_1 (fold Enrichment)** | **upload_1 (raw P-value)** | **upload_1 (FDR)** |
| --- | --- | --- | --- | --- | --- | --- | --- |
| response to hydrogen peroxide (GO:0042542) | 31 | 8 | 0.24 | + | 33.72 | 4.98E-10 | 5.95E-07 |
| protein complex oligomerization (GO:0051259) | 30 | 7 | 0.23 | + | 30.49 | 1.14E-08 | 1.09E-05 |
| response to heat (GO:0009408) | 99 | 13 | 0.76 | + | 17.16 | 3.60E-12 | 1.72E-08 |
| response to reactive oxygen species (GO:0000302) | 65 | 8 | 0.5 | + | 16.08 | 8.71E-08 | 6.93E-05 |
| protein refolding (GO:0042026) | 49 | 5 | 0.37 | + | 13.33 | 5.67E-05 | 2.26E-02 |
| response to temperature stimulus (GO:0009266) | 176 | 15 | 1.35 | + | 11.14 | 2.33E-11 | 5.57E-08 |
| response to salt stress (GO:0009651) | 98 | 8 | 0.75 | + | 10.67 | 1.58E-06 | 8.36E-04 |
| response to osmotic stress (GO:0006970) | 117 | 9 | 0.9 | + | 10.05 | 5.56E-07 | 3.32E-04 |
| protein folding (GO:0006457) | 235 | 16 | 1.8 | + | 8.9 | 1.14E-10 | 1.82E-07 |
| response to inorganic substance (GO:0010035) | 169 | 11 | 1.29 | + | 8.51 | 1.49E-07 | 1.02E-04 |
| response to abiotic stimulus (GO:0009628) | 612 | 18 | 4.68 | + | 3.84 | 1.88E-06 | 8.96E-04 |
| response to stress (GO:0006950) | 1576 | 31 | 12.06 | + | 2.57 | 2.04E-06 | 8.84E-04 |
| response to stimulus (GO:0050896) | 3175 | 45 | 24.3 | + | 1.85 | 6.29E-05 | 2.31E-02 |

**Supplementary Table 2**. GO term enrichment of stem DEGs

GO term enrichment of clusters II and III in Figure 2B

| **GO biological process complete** | **Sorghum bicolor - REFLIST (33714)** | **upload_1 (542)** | **upload_1 (expected)** | **upload_1 (over/under)** | **upload_1 (fold Enrichment)** | **upload_1 (raw P- value)** | **upload_1 (FDR)** |
| --- | --- | --- | --- | --- | --- | --- | --- |
| ammonium transmembrane transport (GO:0072488) | 7 | 3 | 0.11 | + | 26.66 | 4.35E-04 | 4.62E-02 |
| auxin export across the plasma membrane (GO:0010315) | 13 | 5 | 0.21 | + | 23.92 | 7.03E-06 | 2.58E-03 |
| auxin homeostasis (GO:0010252) | 19 | 5 | 0.31 | + | 16.37 | 3.23E-05 | 7.00E-03 |
| auxin polar transport (GO:0009926) | 23 | 5 | 0.37 | + | 13.52 | 7.08E-05 | 1.13E-02 |
| carotene metabolic process (GO:0016119) | 19 | 4 | 0.31 | + | 13.1 | 4.32E-04 | 4.69E-02 |
| regulation of auxin mediated signaling pathway (GO:0010928) | 24 | 5 | 0.39 | + | 12.96 | 8.44E-05 | 1.30E-02 |
| terpene metabolic process (GO:0042214) | 27 | 5 | 0.43 | + | 11.52 | 1.38E-04 | 1.88E-02 |
| hormone transport (GO:0009914) | 36 | 6 | 0.58 | + | 10.37 | 4.94E-05 | 8.73E-03 |
| hydrocarbon metabolic process (GO:0120252) | 31 | 5 | 0.5 | + | 10.03 | 2.45E-04 | 3.25E-02 |
| auxin transport (GO:0060918) | 35 | 5 | 0.56 | + | 8.89 | 4.05E-04 | 4.61E-02 |
| export across plasma membrane (GO:0140115) | 73 | 8 | 1.17 | + | 6.82 | 4.36E-05 | 8.00E-03 |
| auxin-activated signaling pathway (GO:0009734) | 101 | 11 | 1.62 | + | 6.77 | 1.75E-06 | 1.04E-03 |
| cellular response to auxin stimulus (GO:0071365) | 103 | 11 | 1.66 | + | 6.64 | 2.08E-06 | 1.11E-03 |
| amino acid transport (GO:0006865) | 100 | 10 | 1.61 | + | 6.22 | 1.03E-05 | 3.28E-03 |
| organic acid transport (GO:0015849) | 140 | 11 | 2.25 | + | 4.89 | 3.08E-05 | 7.00E-03 |
| regulation of hormone levels (GO:0010817) | 130 | 9 | 2.09 | + | 4.31 | 3.87E-04 | 4.51E-02 |
| response to auxin (GO:0009733) | 209 | 14 | 3.36 | + | 4.17 | 1.46E-05 | 4.34E-03 |
| chemical homeostasis (GO:0048878) | 255 | 14 | 4.1 | + | 3.42 | 1.12E-04 | 1.57E-02 |
| metal ion transport (GO:0030001) | 226 | 12 | 3.63 | + | 3.3 | 4.51E-04 | 4.68E-02 |
| cation transmembrane transport (GO:0098655) | 348 | 18 | 5.59 | + | 3.22 | 2.61E-05 | 6.24E-03 |
| inorganic ion transmembrane transport (GO:0098660) | 337 | 17 | 5.42 | + | 3.14 | 5.84E-05 | 9.61E-03 |
| cation transport (GO:0006812) | 368 | 18 | 5.92 | + | 3.04 | 5.22E-05 | 8.89E-03 |
| homeostatic process (GO:0042592) | 288 | 14 | 4.63 | + | 3.02 | 3.67E-04 | 4.38E-02 |
| inorganic cation transmembrane transport (GO:0098662) | 292 | 14 | 4.69 | + | 2.98 | 4.18E-04 | 4.64E-02 |
| ion transmembrane transport (GO:0034220) | 507 | 24 | 8.15 | + | 2.94 | 5.26E-06 | 2.28E-03 |
| response to external stimulus (GO:0009605) | 424 | 20 | 6.82 | + | 2.93 | 3.32E-05 | 6.88E-03 |
| transmembrane transport (GO:0055085) | 1325 | 60 | 21.3 | + | 2.82 | 2.21E-12 | 1.05E-08 |
| ion transport (GO:0006811) | 626 | 27 | 10.06 | + | 2.68 | 7.07E-06 | 2.41E-03 |
| transport (GO:0006810) | 2411 | 78 | 38.76 | + | 2.01 | 7.96E-09 | 1.90E-05 |
| establishment of localization (GO:0051234) | 2447 | 78 | 39.34 | + | 1.98 | 1.66E-08 | 1.98E-05 |
| localization (GO:0051179) | 2544 | 79 | 40.9 | + | 1.93 | 3.23E-08 | 3.08E-05 |
| biological_process (GO:0008150) | 16640 | 318 | 267.51 | + | 1.19 | 1.74E-05 | 4.61E-03 |
| Unclassified (UNCLASSIFIED) | 17074 | 224 | 274.49 | - | 0.82 | 1.74E-05 | 4.37E-03 |
| cellular nitrogen compound metabolic process (GO:0034641) | 3043 | 21 | 48.92 | - | 0.43 | 6.36E-06 | 2.53E-03 |
| nucleobase-containing compound metabolic process (GO:0006139) | 2241 | 14 | 36.03 | - | 0.39 | 3.48E-05 | 6.93E-03 |
| nucleic acid metabolic process (GO:0090304) | 1852 | 8 | 29.77 | - | 0.27 | 3.37E-06 | 1.61E-03 |
| cellular component biogenesis (GO:0044085) | 1104 | 3 | 17.75 | - | 0.17 | 3.89E-05 | 7.43E-03 |
| gene expression (GO:0010467) | 1664 | 3 | 26.75 | - | 0.11 | 1.39E-08 | 2.22E-05 |
| macromolecule biosynthetic process (GO:0009059) | 1278 | 2 | 20.55 | - | 0.1 | 4.85E-07 | 3.31E-04 |
| regulation of cellular protein metabolic process (GO:0032268) | 685 | 1 | 11.01 | - | 0.09 | 4.84E-04 | 4.91E-02 |
| RNA metabolic process (GO:0016070) | 1393 | 2 | 22.39 | - | 0.09 | 7.25E-08 | 5.77E-05 |
| regulation of protein metabolic process (GO:0051246) | 697 | 1 | 11.21 | - | 0.09 | 3.15E-04 | 4.07E-02 |
| RNA processing (GO:0006396) | 772 | 1 | 12.41 | - | 0.08 | 1.04E-04 | 1.51E-02 |
| cellular macromolecule biosynthetic process (GO:0034645) | 902 | 1 | 14.5 | - | 0.07 | 1.66E-05 | 4.67E-03 |
| peptide biosynthetic process (GO:0043043) | 547 | 0 | 8.79 | - | < 0.01 | 3.53E-04 | 4.32E-02 |
| translation (GO:0006412) | 540 | 0 | 8.68 | - | < 0.01 | 3.39E-04 | 4.26E-02 |
| protein-containing complex organization (GO:0043933) | 515 | 0 | 8.28 | - | < 0.01 | 4.99E-04 | 4.96E-02 |
| amide biosynthetic process (GO:0043604) | 604 | 0 | 9.71 | - | < 0.01 | 1.03E-04 | 1.53E-02 |

GO term enrichment of cluster IV in Figure 2B

| **GO biological process complete** | **Sorghum bicolor - REFLIST (33714)** | **upload_1 (466)** | **upload_1 (expected)** | **upload_1 (over/under)** | **upload_1 (fold Enrichment)** | **upload_1 (raw P- value)** | **upload_1 (FDR)** |
| --- | --- | --- | --- | --- | --- | --- | --- |
| photosystem II stabilization (GO:0042549) | 6 | 3 | 0.08 | + | 36.17 | 1.99E-04 | 1.67E-02 |
| photosystem II repair (GO:0010206) | 8 | 4 | 0.11 | + | 36.17 | 1.55E-05 | 2.17E-03 |
| oxidative photosynthetic carbon pathway (GO:0009854) | 7 | 3 | 0.1 | + | 31.01 | 2.81E-04 | 2.32E-02 |
| phylloquinone metabolic process (GO:0042374) | 10 | 4 | 0.14 | + | 28.94 | 3.06E-05 | 3.85E-03 |
| vitamin K metabolic process (GO:0042373) | 10 | 4 | 0.14 | + | 28.94 | 3.06E-05 | 3.75E-03 |
| phylloquinone biosynthetic process (GO:0042372) | 10 | 4 | 0.14 | + | 28.94 | 3.06E-05 | 3.66E-03 |
| vitamin K biosynthetic process (GO:0042371) | 10 | 4 | 0.14 | + | 28.94 | 3.06E-05 | 3.57E-03 |
| photosystem II assembly (GO:0010207) | 10 | 4 | 0.14 | + | 28.94 | 3.06E-05 | 3.48E-03 |
| regulation of photosynthesis, light reaction (GO:0042548) | 12 | 4 | 0.17 | + | 24.12 | 5.45E-05 | 6.05E-03 |
| plastid translation (GO:0032544) | 9 | 3 | 0.12 | + | 24.12 | 5.05E-04 | 3.83E-02 |
| photosystem I assembly (GO:0048564) | 12 | 4 | 0.17 | + | 24.12 | 5.45E-05 | 5.91E-03 |
| regulation of generation of precursor metabolites and energy (GO:0043467) | 12 | 4 | 0.17 | + | 24.12 | 5.45E-05 | 5.78E-03 |
| glycogen biosynthetic process (GO:0005978) | 10 | 3 | 0.14 | + | 21.7 | 6.50E-04 | 4.85E-02 |

| protoporphyrinogen IX biosynthetic process (GO:0006782) | 14 | 4 | 0.19 | + | 20.67 | 8.97E-05 | 8.92E-03 |
| --- | --- | --- | --- | --- | --- | --- | --- |
| protoporphyrinogen IX metabolic process (GO:0046501) | 14 | 4 | 0.19 | + | 20.67 | 8.97E-05 | 8.74E-03 |
| fat-soluble vitamin metabolic process (GO:0006775) | 15 | 4 | 0.21 | + | 19.29 | 1.12E-04 | 1.07E-02 |
| fat-soluble vitamin biosynthetic process (GO:0042362) | 15 | 4 | 0.21 | + | 19.29 | 1.12E-04 | 1.05E-02 |
| protein repair (GO:0030091) | 15 | 4 | 0.21 | + | 19.29 | 1.12E-04 | 1.03E-02 |
| photorespiration (GO:0009853) | 15 | 4 | 0.21 | + | 19.29 | 1.12E-04 | 1.01E-02 |
| glycogen metabolic process (GO:0005977) | 19 | 5 | 0.26 | + | 19.04 | 1.58E-05 | 2.16E-03 |
| energy reserve metabolic process (GO:0006112) | 19 | 5 | 0.26 | + | 19.04 | 1.58E-05 | 2.10E-03 |
| chlorophyll biosynthetic process (GO:0015995) | 23 | 6 | 0.32 | + | 18.87 | 2.26E-06 | 4.00E-04 |
| regulation of photosynthesis (GO:0010109) | 20 | 5 | 0.28 | + | 18.09 | 1.96E-05 | 2.52E-03 |
| porphyrin-containing compound biosynthetic process (GO:0006779) | 43 | 9 | 0.59 | + | 15.14 | 3.30E-08 | 1.05E-05 |
| tetrapyrrole biosynthetic process (GO:0033014) | 46 | 9 | 0.64 | + | 14.15 | 5.50E-08 | 1.64E-05 |
| photosynthesis, light harvesting (GO:0009765) | 26 | 5 | 0.36 | + | 13.91 | 5.85E-05 | 6.07E-03 |
| chlorophyll metabolic process (GO:0015994) | 40 | 7 | 0.55 | + | 12.66 | 3.29E-06 | 5.41E-04 |
| heme biosynthetic process (GO:0006783) | 23 | 4 | 0.32 | + | 12.58 | 4.67E-04 | 3.71E-02 |
| porphyrin-containing compound metabolic process (GO:0006778) | 64 | 10 | 0.88 | + | 11.3 | 6.64E-08 | 1.76E-05 |
| ketone biosynthetic process (GO:0042181) | 32 | 5 | 0.44 | + | 11.3 | 1.40E-04 | 1.24E-02 |
| quinone biosynthetic process (GO:1901663) | 32 | 5 | 0.44 | + | 11.3 | 1.40E-04 | 1.22E-02 |
| quinone metabolic process (GO:1901661) | 32 | 5 | 0.44 | + | 11.3 | 1.40E-04 | 1.20E-02 |
| photosynthesis, light reaction (GO:0019684) | 97 | 15 | 1.34 | + | 11.19 | 3.92E-11 | 4.68E-08 |
| tetrapyrrole metabolic process (GO:0033013) | 66 | 10 | 0.91 | + | 10.96 | 8.62E-08 | 2.06E-05 |
| protein peptidyl-prolyl isomerization (GO:0000413) | 62 | 9 | 0.86 | + | 10.5 | 5.31E-07 | 1.10E-04 |
| pigment biosynthetic process (GO:0046148) | 66 | 9 | 0.91 | + | 9.87 | 8.54E-07 | 1.70E-04 |
| photosynthesis (GO:0015979) | 222 | 29 | 3.07 | + | 9.45 | 1.71E-18 | 8.18E-15 |
| cellular metabolic compound salvage (GO:0043094) | 43 | 5 | 0.59 | + | 8.41 | 4.87E-04 | 3.81E-02 |
| peptidyl-proline modification (GO:0018208) | 78 | 9 | 1.08 | + | 8.35 | 3.02E-06 | 5.15E-04 |
| pigment metabolic process (GO:0042440) | 82 | 9 | 1.13 | + | 7.94 | 4.40E-06 | 6.99E-04 |
| vitamin biosynthetic process (GO:0009110) | 67 | 6 | 0.93 | + | 6.48 | 4.90E-04 | 3.77E-02 |
| plastid organization (GO:0009657) | 174 | 15 | 2.41 | + | 6.24 | 5.83E-08 | 1.64E-05 |
| chloroplast organization (GO:0009658) | 139 | 11 | 1.92 | + | 5.73 | 7.32E-06 | 1.13E-03 |
| generation of precursor metabolites and energy (GO:0006091) | 436 | 27 | 6.03 | + | 4.48 | 3.44E-10 | 2.74E-07 |
| translation (GO:0006412) | 540 | 28 | 7.46 | + | 3.75 | 6.73E-09 | 3.21E-06 |
| peptide biosynthetic process (GO:0043043) | 547 | 28 | 7.56 | + | 3.7 | 8.76E-09 | 3.80E-06 |
| amide biosynthetic process (GO:0043604) | 604 | 29 | 8.35 | + | 3.47 | 1.81E-08 | 6.64E-06 |
| peptide metabolic process (GO:0006518) | 684 | 30 | 9.45 | + | 3.17 | 6.92E-08 | 1.74E-05 |
| cellular amide metabolic process (GO:0043603) | 793 | 32 | 10.96 | + | 2.92 | 1.54E-07 | 3.35E-05 |
| cellular nitrogen compound biosynthetic process (GO:0044271) | 1265 | 47 | 17.49 | + | 2.69 | 2.13E-09 | 1.45E-06 |
| organonitrogen compound biosynthetic process (GO:1901566) | 1279 | 45 | 17.68 | + | 2.55 | 3.18E-08 | 1.08E-05 |
| cellular macromolecule biosynthetic process (GO:0034645) | 902 | 31 | 12.47 | + | 2.49 | 8.27E-06 | 1.20E-03 |
| cellular biosynthetic process (GO:0044249) | 2405 | 76 | 33.24 | + | 2.29 | 3.02E-11 | 4.80E-08 |
| biosynthetic process (GO:0009058) | 2662 | 84 | 36.79 | + | 2.28 | 3.15E-12 | 7.52E-09 |
| organic substance biosynthetic process (GO:1901576) | 2499 | 77 | 34.54 | + | 2.23 | 7.11E-11 | 6.79E-08 |
| macromolecule biosynthetic process (GO:0009059) | 1278 | 39 | 17.66 | + | 2.21 | 7.81E-06 | 1.16E-03 |
| organic cyclic compound biosynthetic process (GO:1901362) | 867 | 26 | 11.98 | + | 2.17 | 3.32E-04 | 2.69E-02 |
| gene expression (GO:0010467) | 1664 | 49 | 23 | + | 2.13 | 1.57E-06 | 3.00E-04 |
| cellular nitrogen compound metabolic process (GO:0034641) | 3043 | 69 | 42.06 | + | 1.64 | 6.23E-05 | 6.33E-03 |
| metabolic process (GO:0008152) | 9821 | 194 | 135.75 | + | 1.43 | 1.40E-08 | 5.58E-06 |
| cellular metabolic process (GO:0044237) | 8535 | 165 | 117.97 | + | 1.4 | 1.69E-06 | 3.10E-04 |
| cellular process (GO:0009987) | 12229 | 226 | 169.03 | + | 1.34 | 8.64E-08 | 1.96E-05 |
| biological_process (GO:0008150) | 16640 | 294 | 230 | + | 1.28 | 3.47E-09 | 1.84E-06 |
| Unclassified (UNCLASSIFIED) | 17074 | 172 | 236 | - | 0.73 | 3.47E-09 | 2.07E-06 |

| GO term enrichment of cluster V in Figure 2B | | | | | | | |
| --- | --- | --- | --- | --- | --- | --- | --- |
| **GO biological process complete** | **Sorghum bicolor - REFLIST (33714)** | **upload_1 (257)** | **upload_1 (expected)** | **upload_1 (over/under)** | **upload_1 (fold Enrichment)** | **upload_1 (raw P- value)** | **upload_1 (FDR)** |
| protein complex oligomerization (GO:0051259) | 30 | 9 | 0.23 | + | 39.35 | 1.23E-11 | 1.46E-08 |
| response to hydrogen peroxide (GO:0042542) | 31 | 9 | 0.24 | + | 38.09 | 1.57E-11 | 1.50E-08 |
| formaldehyde catabolic process (GO:0046294) | 12 | 3 | 0.09 | + | 32.8 | 1.82E-04 | 4.35E-02 |
| formaldehyde metabolic process (GO:0046292) | 12 | 3 | 0.09 | + | 32.8 | 1.82E-04 | 4.14E-02 |
| cellular response to aldehyde (GO:0110096) | 12 | 3 | 0.09 | + | 32.8 | 1.82E-04 | 3.95E-02 |
| cellular detoxification of aldehyde (GO:0110095) | 12 | 3 | 0.09 | + | 32.8 | 1.82E-04 | 3.78E-02 |
| response to heat (GO:0009408) | 99 | 17 | 0.75 | + | 22.53 | 2.68E-17 | 1.28E-13 |
| response to reactive oxygen species (GO:0000302) | 65 | 9 | 0.5 | + | 18.16 | 5.08E-09 | 3.03E-06 |
| cellular response to heat (GO:0034605) | 32 | 4 | 0.24 | + | 16.4 | 1.56E-04 | 3.91E-02 |
| response to salt stress (GO:0009651) | 98 | 10 | 0.75 | + | 13.39 | 1.04E-08 | 5.53E-06 |
| response to temperature stimulus (GO:0009266) | 176 | 17 | 1.34 | + | 12.67 | 1.52E-13 | 3.64E-10 |
| response to osmotic stress (GO:0006970) | 117 | 11 | 0.89 | + | 12.33 | 4.18E-09 | 2.85E-06 |
| protein folding (GO:0006457) | 235 | 18 | 1.79 | + | 10.05 | 1.13E-12 | 1.80E-09 |
| response to inorganic substance (GO:0010035) | 169 | 11 | 1.29 | + | 8.54 | 1.44E-07 | 6.23E-05 |
| response to oxygen-containing compound (GO:1901700) | 393 | 16 | 3 | + | 5.34 | 1.12E-07 | 5.35E-05 |
| response to oxidative stress (GO:0006979) | 280 | 10 | 2.13 | + | 4.69 | 8.04E-05 | 2.40E-02 |
| response to abiotic stimulus (GO:0009628) | 612 | 18 | 4.67 | + | 3.86 | 1.78E-06 | 7.07E-04 |

| protein-containing complex assembly (GO:0065003) | 449 | 12 | 3.42 | + | 3.51 | 2.28E-04 | 4.53E-02 |
| --- | --- | --- | --- | --- | --- | --- | --- |
| cellular response to organic substance (GO:0071310) | 449 | 12 | 3.42 | + | 3.51 | 2.28E-04 | 4.35E-02 |
| response to chemical (GO:0042221) | 1222 | 32 | 9.32 | + | 3.44 | 2.18E-09 | 1.73E-06 |
| response to organic substance (GO:0010033) | 755 | 18 | 5.76 | + | 3.13 | 2.83E-05 | 9.66E-03 |
| cellular response to chemical stimulus (GO:0070887) | 739 | 17 | 5.63 | + | 3.02 | 7.25E-05 | 2.31E-02 |
| response to stress (GO:0006950) | 1576 | 27 | 12.01 | + | 2.25 | 1.46E-04 | 3.88E-02 |
| response to stimulus (GO:0050896) | 3175 | 47 | 24.2 | + | 1.94 | 1.36E-05 | 5.01E-03 |
| macromolecule metabolic process (GO:0043170) | 6529 | 26 | 49.77 | - | 0.52 | 9.01E-05 | 2.53E-02 |

**Supplementary Table 3**. Transcript levels of intersection genes of different factors in stem

Transcript levels of DEGs in the center of Venn diagram shown in Fig. 3B.

Transcript levels are presented as transcript per million (TPM). The TPM value is mean ± SE (n = 3 or 4).

**Supplementary Table 4**. Transcript levels (TPM) of starch and PCD related genes under different conditions.

| **Gene ID** | **Gene Name** | **DD_3 day** | | **DD_10 day** | | **dd_3 day** | | **dd_10 day** | |
| --- | --- | --- | --- | --- | --- | --- | --- | --- | --- |
|  |  | **Ctrl** | **HR** | **Ctrl** | **HR** | **Ctrl** | **HR** | **Ctrl** | **HR** |
| Sobic.007G204600 | *Isoamylase 1* | 17.29 | 32.40 | 12.79 | 51.49 | 18.66 | 40.71 | 14.49 | 96.39 |
| Sobic.001G083900 | *Starch phosphorylase a* | 66.73 | 151.65 | 75.13 | 221.84 | 103.22 | 188.64 | 82.84 | 457.03 |
| Sobic.003G358600 | *Starch phosphorylase b* | 118.06 | 194.53 | 199.61 | 234.13 | 200.56 | 270.33 | 146.43 | 309.53 |
| Sobic.007G101500 | *AGPase small subunit a* | 65.63 | 83.88 | 60.33 | 99.59 | 79.57 | 118.03 | 69.82 | 148.84 |
| Sobic.002G160400 | *AGPase small subunit b* | 102.65 | 167.23 | 108.86 | 240.26 | 140.95 | 256.96 | 134.18 | 335.87 |
| Sobic.001G100000 | *AGPase large subunit* | 13.74 | 22.64 | 11.68 | 22.91 | 15.54 | 33.55 | 13.40 | 40.95 |
| Sobic.002G233600 | *Isoamylase 3* | 52.91 | 68.24 | 43.93 | 61.14 | 62.65 | 87.97 | 50.53 | 86.95 |
| Sobic.010G143500 | *Alpha-glucan dikinase* | 49.96 | 92.14 | 63.26 | 94.11 | 54.33 | 97.34 | 55.14 | 103.70 |
| Sobic.010G273800 | *starch branching enzyme 2.2a* | 112.95 | 250.91 | 137.32 | 366.78 | 137.55 | 310.45 | 150.07 | 400.02 |
| Sobic.010G047700 | *Starch synthase 1* | 106.40 | 148.58 | 105.29 | 207.03 | 270.67 | 286.46 | 201.17 | 361.04 |
| Sobic.003G084000 | *SbpGlct* | 174.39 | 227.40 | 184.83 | 197.85 | 204.33 | 286.78 | 181.29 | 210.35 |
| Sobic.007G065500 | *SbpGPT1* | 191.79 | 167.69 | 224.20 | 147.83 | 163.88 | 108.05 | 159.47 | 131.35 |
| Sobic.006G147400 | *SbNAC074A* | 21.60 | 12.54 | 6.30 | 0.91 | 24.83 | 22.78 | 2.61 | 0.89 |
| Sobic.008G020700 | *SbMIF* | 314.22 | 165.98 | 263.24 | 118.82 | 223.78 | 138.01 | 338.40 | 99.15 |
| Sobic.007G172100 | *SbXCP1* | 51.94 | 33.59 | 12.00 | 1.92 | 0.13 | 0.02 | 0.15 | 0.18 |
| Sobic.003G087200 | *BFN1 homolog* | 150.71 | 119.34 | 195.42 | 73.19 | 187.58 | 190.58 | 202.26 | 64.77 |
| Sobic.004G187200 | *Type II metacaspase* | 2.12 | 1.24 | 6.24 | 1.12 | 1.91 | 0.96 | 2.52 | 0.97 |
| Sobic.004G010000 | *SCPL48 homolog* | 6.39 | 5.10 | 4.33 | 0.85 | 3.34 | 0.57 | 1.69 | 0.75 |

TPM, transcript per million. The mean TPM value (n = 3 or 4) was shown in each cell under that condition.

Apply a color gradient (white-red) to a range of cells (minimum-maximum). The background color indicates where each cell value falls within that range.

**Supplementary Table 5.** Effects of treatment, genotype and day on transcript levels of sugar transporter genes.

| **Gene Name** | **Gene ID** | **Stem** | | | | | | **Leaf** | | | | | |
| --- | --- | --- | --- | --- | --- | --- | --- | --- | --- | --- | --- | --- | --- |
|  |  | **Treatment** | | **Genotype** | | **Day** | | **Treatment** | | **Genotype** | | **Day** | |
|  |  | **Effect size** | **p adj** | **Effect size** | **p adj** | **Effect size** | **p adj** | **Effect size** | **p adj** | **Effect size** | **p adj** | **Effect size** | **p adj** |
| SbSWEET13A | Sobic.008G094000 | 0.47 | 0.0045 | -0.38 | 0.0099 | 0.02 | 0.8875 | -0.15 | 0.1687 | -0.01 | 0.9068 | -0.30 | 0.0028 |
| SbSUT1 | Sobic.001G488700 | 0.36 | 0.0046 | -0.40 | 0.0019 | -0.67 | 1.68E-06 | -0.13 | 0.1001 | -0.19 | 0.0182 | -0.78 | 2.84E-26 |
| SbSWEET1A | Sobic.003G377700 | 1.56 | 1.86E-15 | -0.11 | 0.4075 | 1.36 | 9.58E-12 | 0.09 | 0.5911 | 0.02 | 0.8823 | -0.19 | 0.2575 |
| SbSWEET16 | Sobic.001G377600 | -0.28 | 0.1881 | -0.22 | 0.0952 | 0.02 | 0.8950 | 0.29 | 0.1005 | -0.21 | 0.1482 | -0.04 | 0.8331 |
| SbSWEET3A | Sobic.009G080900 | -0.36 | 0.1153 | -0.02 | 0.8728 | 0.44 | 0.0471 | 0.29 | 0.0492 | 0.04 | 0.7948 | 0.55 | 0.0008 |
| SbSWEET4A | Sobic.004G136600 | -1.06 | 3.86E-09 | -0.52 | 0.0011 | -0.30 | 0.0368 | -0.19 | 0.1812 | -0.48 | 0.0012 | -0.28 | 0.0522 |

Estimated effect size under a linear model in DESeq2 is equivalent to the log2FC in a pairwise comparison

**Supplementary Table 6.** Sequences of oligonucleotide used in this study

| **Name** | **Sequence 5’-3’** | **Purpose** |
| --- | --- | --- |
| SbSWEET1A-T7F | TAATACGACTCACTATAGGGTACCGAAAAAACAAGGGCC | anti-sense probe |
| SbSWEET1A-R | TAGCAGTTGGATCAATTCCTC | anti-sense probe |
| SbSWEET1A-F | TACCGAAAAAACAAGGGCC | sense probe |
| SbSWEET1A-T7R | TAATACGACTCACTATAGGGTAGCAGTTGGATCAATTCCTC | sense probe |
| SbCINV5-SP6-F | ATTTAGGTGACACTATAGAACCTCCTTTTATCCCCTCTCG | anti-sense probe |
| SbCINV5-R | ATTGCCACCTACAGCCTGAC | anti-sense probe |
| SbCINV5-F | CCTCCTTTTATCCCCTCTCG | sense probe |
| SbCINV5-SP6-R | ATTTAGGTGACACTATAGAAATTGCCACCTACAGCCTGAC | sense probe |

**
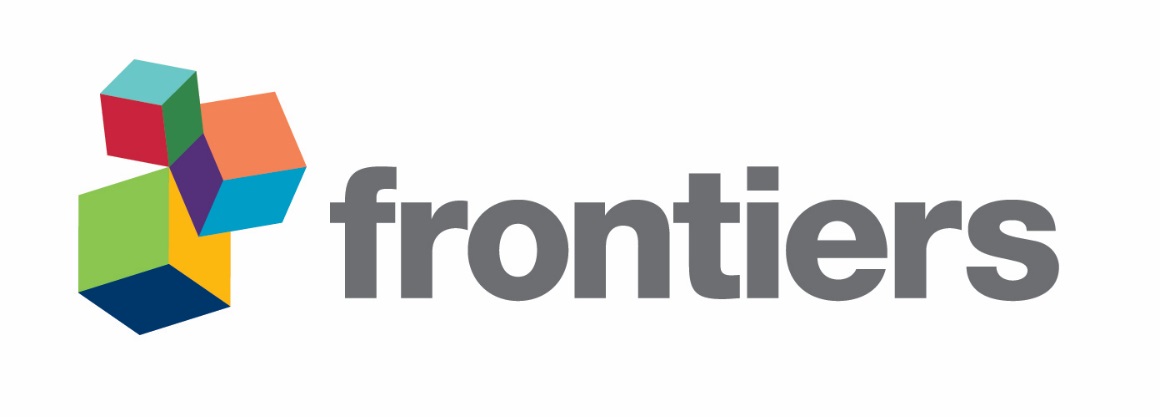
**
